# Exploitation of surrogate variables in random forests for unbiased analysis of mutual impact and importance of features

**DOI:** 10.1093/bioinformatics/btad471

**Published:** 2023-07-31

**Authors:** Lucas F Voges, Lukas C Jarren, Stephan Seifert

**Affiliations:** Centre for the Study of Manuscript Cultures (CSMC), Universität Hamburg, Hamburg 20354, Germany; Centre for the Study of Manuscript Cultures (CSMC), Universität Hamburg, Hamburg 20354, Germany; Hamburg School of Food Science, Institute of Food Chemistry, University of Hamburg, Hamburg 20146, Germany; Centre for the Study of Manuscript Cultures (CSMC), Universität Hamburg, Hamburg 20354, Germany; Hamburg School of Food Science, Institute of Food Chemistry, University of Hamburg, Hamburg 20146, Germany

## Abstract

**Motivation:**

Random forest is a popular machine learning approach for the analysis of high-dimensional data because it is flexible and provides variable importance measures for the selection of relevant features. However, the complex relationships between the features are usually not considered for the selection and thus also neglected for the characterization of the analysed samples.

**Results:**

Here we propose two novel approaches that focus on the mutual impact of features in random forests. Mutual forest impact (MFI) is a relation parameter that evaluates the mutual association of the features to the outcome and, hence, goes beyond the analysis of correlation coefficients. Mutual impurity reduction (MIR) is an importance measure that combines this relation parameter with the importance of the individual features. MIR and MFI are implemented together with testing procedures that generate *P*-values for the selection of related and important features. Applications to one experimental and various simulated datasets and the comparison to other methods for feature selection and relation analysis show that MFI and MIR are very promising to shed light on the complex relationships between features and outcome. In addition, they are not affected by common biases, e.g. that features with many possible splits or high minor allele frequencies are preferred.

**Availability and implementation:**

The approaches are implemented in Version 0.3.3 of the R package RFSurrogates that is available at github.com/AGSeifert/RFSurrogates and the data are available at doi.org/10.25592/uhhfdm.12620.

## 1 Introduction

The application of the machine learning approach random forest (RF, Breiman 2001) has become very popular for the analysis of high-dimensional data, e.g. generated in genomics (Chen and Ishwaran 2012) and metabolomics (Chen *et al.* 2013) experiments or genome-wide association studies (GWAS) (Nicholls *et al.* 2020). The reason for this popularity are specific advantages over other methods, such as the flexibility in terms of input and output variables, since both quantitative and qualitative variables can be used to build classification, regression (Strobl *et al.* 2009), and survival models (Ishwaran *et al.* 2011). Another advantage is the ability to generate variable importance measures (VIMs) that are utilized to select the relevant features for parsimonious prediction models or to identify and interpret differences between the samples.

The most common VIMs are the permutation importance and the impurity importance, so called since it is calculated from the impurity gain that the variable contributes to the RF. Another importance measure is minimal depth, which is based on the position of the variables in the decision trees (Ishwaran *et al.* 2010). Minimal depth and the impurity importance, however, are known to be biased in favour of features with many categories (Strobl *et al.* 2007) and high category frequencies (Nicodemus 2011), which is particularly important in GWAS (Boulesteix *et al.* 2012). Since it is not affected by these biases, the permutation importance has been preferred and various selection techniques based on this importance measure have been developed (Kursa and Rudnicki 2010, Szymczak *et al.* 2016, Janitza *et al.* 2018) and compared (Degenhardt *et al.* 2019). A few years ago, a corrected, unbiased impurity importance measure, the actual impurity reduction (AIR), was introduced (Nembrini *et al.* 2018). AIR is computed faster than the permutation importance, which is why this importance measure is very useful for application to high-dimensional data.

All of the RF based importance measures are affected by the correlation structure of the features (Nicodemus *et al.* 2010), and conditional variable importance has been proposed to determine the corrected, individual impact of the features (Strobl *et al.* 2008, Debeer and Strobl 2020). We have taken a different approach to this issue because we think that the relations between the features should be included into the analysis treating them as interacting components. Therefore, we have deliberately included feature relations to improve importance calculation, the power of feature selection and interpretation of differences between samples (Seifert *et al.* 2019). We have achieved this by the exploitation of surrogate variables that have been introduced to compensate for missing values in the data, representing the features that can replace another feature in a split as best as possible (Breiman *et al.* 1984).

Based on surrogate variables, we developed Surrogate Minimal Depth (SMD), an importance measure that incorporates surrogate variables into the concept of minimal depth, and mean adjusted agreement, a relation parameter that is determined by the split agreement of the features across the RF. Since this relation parameter considers the mutual impact of the features on the RF model, this parameter goes beyond the analysis of ordinary correlation coefficients enabling a comprehensive analysis of the complex interplay of features and outcome. We applied this relation analysis to reveal relations between features in gene expression (Seifert *et al.* 2019), metabolomics (Wenck *et al.* 2022), and various spectroscopic datasets (Seifert 2020, Shakiba *et al.* 2022), e.g. to illuminate the interaction of drugs with proteins and lipids in living cells (Zivanovic *et al.* 2019). However, since both SMD and mean adjusted agreement are affected by the previously described biases, their application has so far been limited. Here we introduce two novel approaches for the analysis of feature relations and mutual importance called mutual forest impact (MFI) and mutual impurity reduction (MIR). We will show that they are not affected by these biases and compare their performance with existing approaches by applying them to one experimental and different simulated datasets.

## 2 Materials and methods

### 2.1 Random forest

RF is an ensemble of binary decision trees for classification, regression, and survival analysis (Breiman 2001). Each of these decision trees is built from a different bootstrap sample and the best split for each node is identified from randomly chosen candidate features by maximizing the decrease of impurity. The impurity reduction is usually determined by the Gini index for classification (Breiman *et al.* 1984), the sum of squares for regression (Ishwaran 2015), and the log-rank statistic for survival analysis (Ishwaran *et al.* 2008). The three most important parameters of RF are *num.trees*, the total number of decision trees, *mtry*, the number of randomly chosen candidate features per split, and *min.node.size*, the minimum number of samples for which a further split is made. Since RF is based on bagging, there are out-of-bag samples for each tree, which have not been used in the training process and therefore can be utilized to determine prediction accuracy and variable importance.

### 2.2 Impurity importance

The impurity importance is based on the decrease of impurity determined by the difference of a node’s impurity and the weighted sum of the child node’s impurities. To determine the importance of a feature, the sum of all impurity decrease measures of the nodes based on this feature is divided by the number of trees. The impurity importance is biased in favour of features with many possible split points because they have a higher probability to be randomly suitable for the intended distinction (Strobl *et al.* 2007, Wright *et al.* 2017). In addition, features with the same number of categories but different category frequencies are biased as well (Nicodemus 2011), which is crucial for genetic analyses because single-nucleotide polymorphisms (SNPs) with high minor allele frequencies (MAFs) are favoured (Boulesteix *et al.* 2012). In order to correct this bias, Nembrini *et al.* (2018) applied a modified approach of Sandri and Zuccolotto (2008) to calculate the AIR of each feature *X_i_* by the difference between the VIM and the VIM of a permuted version of itself:


$$\hat{AIR_{X_{i}}}=\hat{VIM_{X_{i}}}-\hat{VIM_{X_{i,\mathrm{perm}}}}$$

### 2.3 Surrogate variables for relation analysis

Surrogate variables were introduced by Breiman *et al.* (1984) for the compensation of missing values. The basic idea is that in addition to the primary splits of the decision trees in RF, alternative splits are determined based on other features of the data that can replace the primary split as best as possible. For the selection of the predefined number *s* of surrogate splits, the surrogates with the highest values for the adjusted agreement *adj* are chosen. This parameter is calculated for the primary split *p* and the possible surrogate *q* utilizing the agreement *n_surr_*, which is determined by the number of samples that are assigned to the same daughter nodes. It is defined by where *n_total_* is the total number of samples at the respective node and *n_maj_* is the number of correct assignments when all samples are assigned to the daughter node with the larger number of samples, also called the majority rule. Note that surrogate variables are only chosen when the adjusted agreement is higher than zero meaning that the surrogate split has to outperform the majority rule. This can result in less than *s* surrogate splits for individual nodes.


$$\mathrm{adj}(p,q)=\frac{n_{\mathrm{surr}}-n_{\mathrm{maj}}}{n_{\mathrm{total}}-n_{\mathrm{maj}}}$$

For the analysis of the variable relations of *X_j_* to *X_i_*, all nodes with primary split variable *X_i_* are considered and the mean adjusted agreement $M_{X_{i}X_{j}}$ is defined by with $p_{n}^{X_{i}}$ and $q_{n}^{X_{j}}$ denoting the primary split based on variable *X_i_* and the surrogate split on variable *X_j_* of the node *n* and $\mathrm{nodes}(X_{i})$ denoting the total number of nodes based on *X_i_*. Related features can subsequently be selected by a threshold adjusted by a user defined factor *t* (default = 5).


$$M_{X_{i}X_{j}}=\frac{\sum_{n=1}^{\left|\mathrm{nodes}(X_{i})\right|}adj(p_{n}^{X_{i}},q_{n}^{X_{j}})}{\left|\mathrm{nodes}(X_{i})\right|}$$

### 2.4 Mututal forest impact

Inspired by Nembrini *et al.* (2018), we developed an unbiased approach for the analysis of variable relations based on surrogate variables. For this purpose, pseudo data *Z*, which is uninformative but shares the structure of the original data *X*, is generated by the permutation of the features across observations. Thus, *Z* contains as many permuted variables *p* as *X*. Subsequently, the mean adjusted agreement of the features is determined for both, *X* and *Z* and the novel relation parameter MFI is defined by


$$\hat{MFI_{X_{i},X_{j}}}=\hat{M_{X_{i},X_{j}}}-\hat{M_{Z_{i},Z_{j}}}$$

Just as for the mean adjusted agreement, a value of one for the MFI of two features corresponds to an exact agreement of their impact on the RF model.

### 2.5 Mutual impurity reduction

Not only the relation parameter but also the importances obtained by SMD are biased. For this reason, we define the novel, unbiased importance measure MIR that does not evaluate the features individually but also considers the relations between them. MIR is the sum of the AIR of the individual feature and the AIR of the other features multiplied by the corresponding relation parameter MFI:


$$\hat{MIR_{X_{i}}}=\hat{AIR_{X_{i}}}+\sum_{j}^{p}\hat{MFI_{X_{i},X_{j}}}\cdot \hat{AIR_{X_{j}}}$$

### 2.6 Testing procedures

Also inspired by Nembrini *et al.* (2018), statistical testing procedure is performed to select relevant and related parameters and the following null hypotheses are tested:


$$H_{0}:MFI_{X_{i},X_{j}}\leq 0\;\;\;\text{and}\;\;\;H_{0}:MIR_{X_{i}}\leq 0$$

For this, the respective importance and relation values are tested against a null distribution. This null distribution is obtained by zero and negative values, which are mirrored to obtain the corresponding positive values, as proposed by Janitza *et al.* (2018). However, for this approach to be validly applied, there must be a sufficient number of negative values and thus a sufficient number of features *p*. Because of this, we developed alternative, permutation-based approaches to obtain the null distributions for important and related features. For the selection of related features in MFI, the permuted relations $\hat{M_{Z_{i}}}$ are utilized. Since they only contain zero and positive values, the null distribution is completed similarly as in Janitza *et al.* (2018): the non-zero values are mirrored to obtain the respective negative values of the distribution. For the selection of important features in MIR, the permuted relations $\hat{M_{Z}}$ are multiplied by permuted values of AIR. This process is repeated multiple times to determine enough values to describe the null distribution sufficiently.

### 2.7 Implementation and analyses

We implemented the newly developed approaches MFI and MIR in R version 3.6.3 (R Core Team 2022) and added them to our package SurrogateMinimalDepth, which was therefore renamed to RFSurrogates. (github.com/AGSeifert/RFSurrogates). In addition to this package, we used ranger (Wright and Ziegler 2017) for the generation of RFs and to obtain AIR values (importance = ‘impurity_corrected’) and select features using the importance_pvalues function.

For the run time investigation, a workstation with 2 IntelXeon Gold 5220R 2.2 GHz processors, 48 cores, and 384 GB DDR4 RAM was used.

### 2.8 Simulation studies

Supplementary Table S1 gives a brief overview over the conducted simulation studies according to the ADEMP scheme (Morris *et al.* 2019). The code to generate the simulated data and the real data analysed are published at doi.org/10.25592/uhhfdm.12620.

#### 2.8.1 Bias of importance and relation measures

Building on previous simulation studies (Strobl *et al.* 2007, Boulesteix *et al.* 2012, Nembrini *et al.* 2018), we simulated two simple null scenarios, meaning that none of the simulated variables is associated with the outcome, to study the bias of the SMD importance and relation analysis. For both, the sample size was set to 100 and the simulation was replicated 1000 times. The outcomes for classification, regression, and survival analyses were generated from a binomial distribution with a probability of 0.5, a standard normal distribution and an exponentially distributed survival (*λ*  =  0.5) and censoring time (*λ* = 0.1), respectively.

AIR, SMD importance and relation analysis, as well as the new approaches MFI and MIR were applied to the simulated data utilizing RF with 100 trees, an *mtry* of three, and a minimal node size of one, three, and five for classification, survival, and regression analysis, respectively. Furthermore, for SMD, MFI, and MIR three surrogates *s* were determined in each split. The variables of the two scenarios were simulated as follows:

*Null scenario A: increasing number of expression possibilities*: Nine nominal variables *X*_1_,…*X*_9_ with 2, 3, 4, 5, 6, 7, 8, 10, and 20 categories were generated from a uniform distribution and one continuous variable *X*_10_ from a standard normal distribution.

*Null scenario B: increasing minor allel frequency*: As in Nembrini *et al.* (2018), 10 variables *X*_1_, …, *X*_10_ with minor allele frequencies of 0.05, 0.10, 0.15, 0.20, 0.25, 0.30, 0.35, 0.40, 0.45, and 0.50 from a binomial distribution were simulated.

#### 2.8.2 Correlation study

To evaluate the selection of variables in the presence of correlations, a simulation study from Seifert *et al.* (2019) was carried out. The quantitative outcome *Y* is dependent on six relevant variables *X*_1_, …, *X*_6_ that were, just like three non-relevant, outcome-independent variables *X*_7_, …, *X*_9_, sampled from a standard normal distribution *N*(0,1):


$$Y=X_{1}+X_{2}+X_{3}+X_{4}+X_{5}+X_{6}+\epsilon$$

The noise *ϵ* followed a *N*(0, 0.2) distribution. In addition, 10 correlated variables (denoted as c*X*_1_, c*X*_2_, c*X*_3_, c*X*_7_, c*X*_8_, and c*X*_9_) were generated for each of *X*_1_, *X*_2_, *X*_3_, *X*_7_, *X*_8_, and *X*_9_ utilizing the simulateModule function of the R package WGCNA (Langfelder and Horvath 2008). This function creates modules of variables with defined correlations around a prespecified variable. Strong correlations (0.9) were used for *X*_1_ and *X*_7_, medium correlations (0.6) for *X*_2_ and *X*_8_, and low correlations (0.3) for *X*_3_ and *X*_9_. Furthermore, to reach a total number of *p*_1_ = 1000 variables, additional independent, non-correlated variables (ncV) were simulated using a standard normal distribution. We will denote the variables *X*_1_, …, *X*_6_ as well as c*X*_1_, c*X*_2_, and c*X*_3_ as relevant and the other variables as non-relevant. For a graphical summary of the simulation, refer to Supplementary material in Seifert *et al.* (2019).

We simulated data for 100 individuals, generated 100 replicates and applied the importance measures and feature selection approaches AIR, SMD, and MIR utilizing RFs with 1000 trees, an *mtry* of 177 (^=  $p_{1}^{\left(3/4\right)}$), and a minimal node size of five. For AIR and MIR, the *P*-value thresholds .001, .01, and .05 and for SMD and MIR, *s* values of 5, 10, 20, and 100 were used.

In order to compare type I error rates, we also simulated a null scenario with 1000 independent predictor variables from a standard normal distribution and an independent binary outcome. Fifty replicates for 50 cases and 50 controls were simulated and AIR, MIR, and SMD were applied with the same parameters as above but a minimal node size of one. Type I error rates were subsequently estimated by the division of selected variables and the total number of predictor variables.

#### 2.8.3 Realistic study

For the comparison of the feature selection approaches under realistic correlation structures, gene expression datasets were simulated as in Seifert *et al.* (2019) and Degenhardt *et al.* (2019) utilizing the R package Umpire (Zhang *et al.* 2012) and a realistic covariance matrix. The covariance matrix was generated from an RNA-microarray dataset of breast cancer patients with *p*_2_ = 12 592 genes obtained from The Cancer Genome Atlas Network (2012). A multivariate normal distribution for a 12 592-dimensional random vector with a mean vector of zeroes was applied to obtain gene expression values for cases and controls. For the cases, 25 variables for each effect size from the set {−2,−1,−0.5,0.5,1,2} were randomly chosen and the means of the variables were increased accordingly. The other variables and the covariance matrix were the same for cases and controls. Two datasets with 100 controls and 100 cases were simulated for each set of 150 causal variables and the whole process including random selection of causal variables was repeated 50 times.

As in Seifert *et al.* (2019), we estimated the evaluation criteria stability, classification error, empirical power, and false positive rate (FPR) for the comparison of the performance of AIR, MIR, and SMD. The approaches were applied using RFs with 5000 trees, an *mtry* of 1188 (^=$p_{2}^{\left(3/4\right)}$), and a minimal node size of one. For AIR and MIR, a *P*-value threshold of .01 and for SMD and MIR *s* values of 50, 100, 200, and 500 were used.

Stability was determined by the Jaccard’s index (He and Yu 2010), the ratio of the length of the intersection and the length of the union of the two sets of selected variables. The classification error was determined using each of the two datasets of each replicate to select variables, on which RF models with the same parameters as for the feature selection were trained. The other dataset was subsequently used as validation set and the mean classification error was calculated for each pair. The empirical power was determined separately for each absolute effect size by the fraction of correct selections among all replicates. To determine the FPR, the null variables were defined differently for each replicate characterized by other causal variables. Since variables at least moderately correlated to causal variables are of interest as well, only non-causal and ncV (correlation coefficient <0.2) were defined as null variables. The FPR was calculated by dividing the number of selected null variables by the total numbers of null variables.

### 2.9 Real data application

To apply feature selection and relation analysis on real experimental data with categorical variables, we used single-nucleotide variations (SNV/SNP) of a *Solanum* Section *Petota* species plastid genome dataset licensed under CC BY 4.0 (creativecommons.org/licenses/by/4.0/) from Huang *et al.* (2019); a subset of the original data was used (see Table 1). For SNP identification, multiple sequence alignments of 43 genes were conducted with QIAGEN CLC Genomics Workbench 22.0.2 (digitalinsights.qiagen.com) and SNP-sites 2.5.1 (Page *et al.* 2016) was subsequently used to generate variant call format files. These files were merged into a file of $p_{3}=257$ SNPs for further analysis in R.

**Table 1.** Selected plastid DNA samples of *Solanum* Section *Petota* species with corresponding provenance from Huang *et al.* (2019).

	Species	No. samples	Country
1	*canasense*	11	Peru/Bolivia
2	*gourlayi*	12	Argentina/Bolivia
3	*verrucosum*	9	Mexico

RFs for the classification of species and provenance were trained using a minimal node size of one, an *mtry* of 198 (^=$p_{3}^{\left(3/4\right)}$) and 1000 trees. For MIR and AIR, permuted variables were utilized to compute *P*-values for feature selection and a *P*-value threshold of .01 was applied. For MIR, *s* values of 3, 8, 13, 18, 26, and 52 corresponding to 1%, 3%, 5%, 7%, 10%, and 20% of *p*_3_ were used. Results were reported using the R packages ggplot2 3.4.1 (Wickham 2016), VennDiagram 1.7.3 (Chen and Boutros 2011), cowplot 1.1.1 (Wilke 2020), and pheatmap 1.0.12 (Kolde 2019).

## 3 Results

### 3.1 Simulation studies

#### 3.1.1 Bias of relation measures

**Figure 1.**
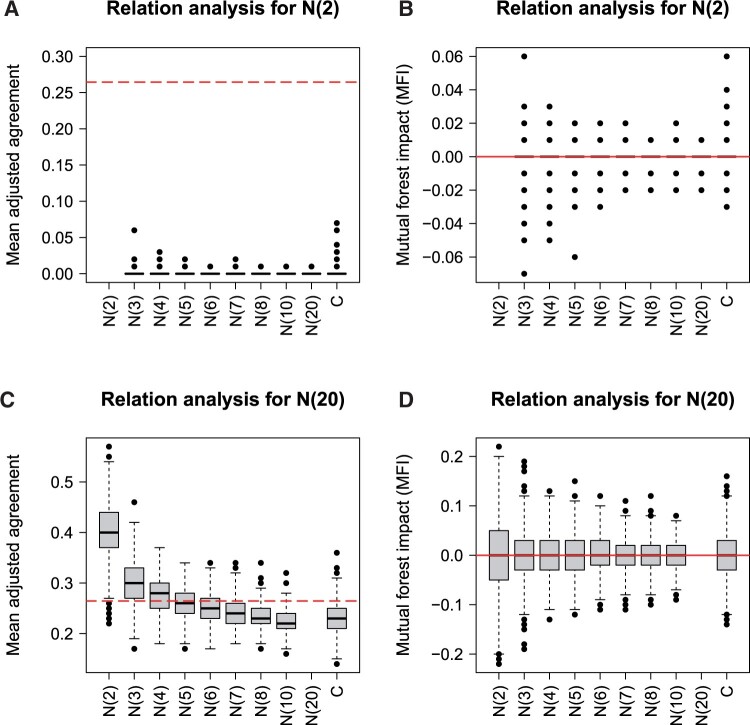
Null scenario A (classification): relation analysis based on mean adjusted agreement (A and C) and MFI (B and D) for the nominal variables with 2 (A and B) and 20 (C and D) categories. The red line shows the threshold for the selection of related variables and a relation value of zero for mean adjusted agreement (*t* = 5) and MFI, respectively. The respective missing values result from the non-existing relations of variables with themself and the relations of the other variables are shown in Supplementary Figs S1 and S2.

The classification results for the null scenario with increasing number of expression possibilities (null scenario A) are shown in Fig. 1. The mean adjusted agreement is zero or very close to zero, when the relations of the variable with two categories are analysed (Fig. 1A). Since all these values are below the threshold, no variable is falsely selected here. However, this does not apply to the relation analysis of the variable with 20 categories (Fig. 1C). Because this variable has much more expression possibilities, it shows increasing values for the mean adjusted agreement as the number of categories decrease. For the variable with only two categories, quite high values of ∼0.4 are obtained resulting in a very frequent false selection of the relation between these two variables. It is obvious that, similar as for the importance analysis (Strobl *et al.* 2007), variables with many categories are favoured in the relation analysis, especially when relations to variables with low numbers of categories are analysed. MFI, the novel approach for relation analysis, does not show this bias, since all values for both, the variable with 2 and 20 categories, are located around zero (Fig. 1B and D). For the latter, however, the variance increases for variables with lower numbers of categories.

**Figure 2.**
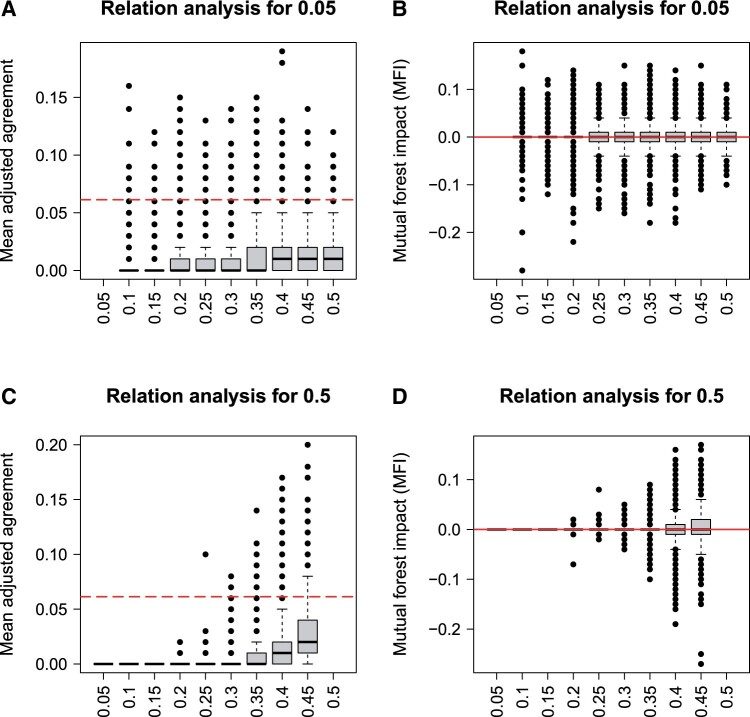
Null scenario B (classification): relation analysis based on mean adjusted agreement (A and C) and MFI (B and D) for the variables with minor allele frequencies of 0.05 (A and B) and 0.5 (C and D). The line shows the threshold for the selection of related variables and a relation value of zero for mean adjusted agreement (*t* = 5) and MFI, respectively. The respective missing values result from the non-existing relations of variables with themself and the relations of the other variables are shown in Supplementary Figs S3 and S4.

Figure 2 shows the results for the analysis of increasing MAF. The mean adjusted agreement of variables with higher MAF is generally higher for the relation analysis of both, the variable with a low MAF of 0.05 (Fig. 2A) and the variable with a high MAF of 0.5 (Fig. 2C). Consequently, relations of variables with high MAF are falsely selected more frequently. The MFI is not influenced by the MAF in the same way, because all variables show values around zero for both variables (Fig. 2B and D). However, the variance increases for variables with higher MAF. The bias analysis of mean adjusted agreement and MFI, which is shown here for a classification outcome, is also reflected in the regression and survival analyses (see Supplementary Figs S5–S12).

#### 3.1.2 Bias of importance measures

**Figure 3.**
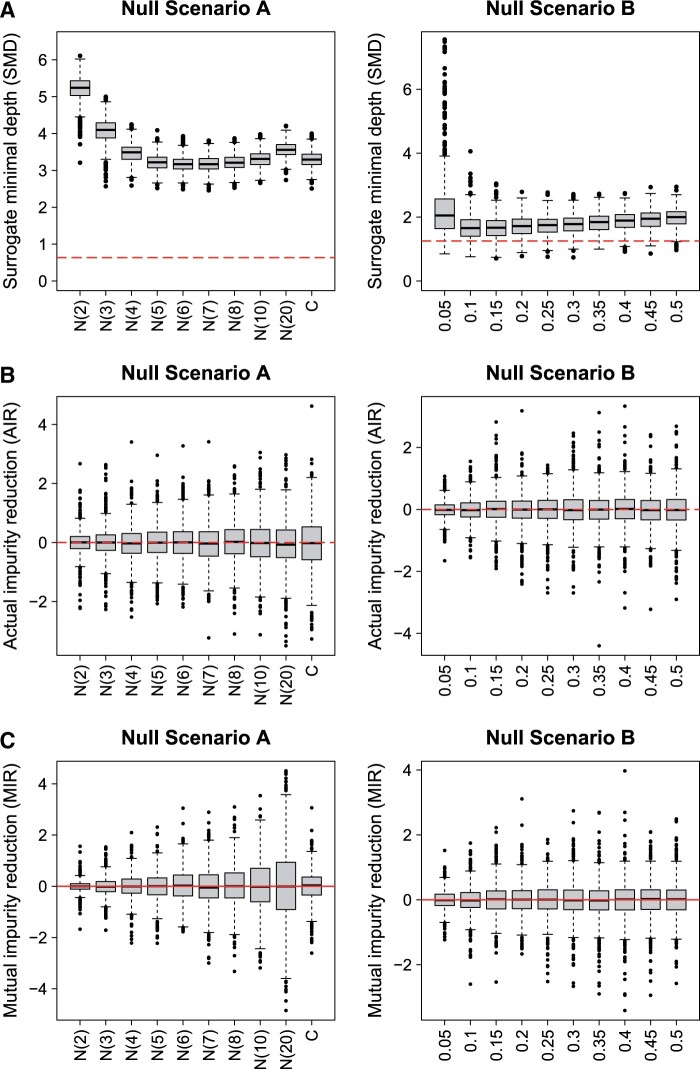
Variable importances for null scenarios (classification): results of SMD (A), AIR (B), and MIR (C) in null scenario A (left) and B (right). The red line shows the threshold for the feature selection and an importance value of zero for SMD (*t* = 5) and AIR and MIR, respectively. Note that low SMD values correspond to important features.

The results for the importance analyses for both null scenarios are shown in Fig. 3. SMD shows importance scores dependent on the number of categories: (Fig. 3A left) variables with low numbers of categories have high values for SMD corresponding to low importances, while variables with high numbers of categories seem more important because they have lower SMD values. Hence, SMD shows the same bias as other importance measures favouring variables with many categories (Strobl *et al.* 2007). MIR, just like AIR, does not show this bias (Fig. 3B and C left). However, the well-known property of higher variances for variables with higher numbers of categories can be observed for AIR (Nembrini *et al.* 2018). For MIR, this influence is even more evident.

SMD is also influenced by the MAF showing different importance values for variables with different MAF and consequently also different numbers of false selections (Fig. 1A right). For AIR and MIR, this bias is not observed. However, as expected from Nembrini *et al.* (2018), the variance of AIR increases towards higher MAF. For MIR, the same property is apparent.

#### 3.1.3 Correlation study

**Figure 4.**
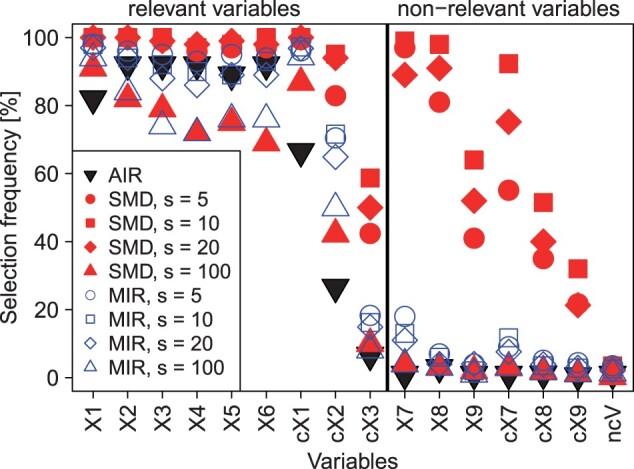
Results of the correlation study utilizing different numbers of surrogate variables (SMD and MIR) and a *P*-value thresholds of .01 (AIR and MIR). For the basic variables, (*X*_1_–*X*_9_) the selection frequencies are averaged across all 50 replicates, whereas for the six groups of correlated variables (c*X*_1_–c*X*_3_ and c*X*_7_–c*X*_9_) as well as the non-causal variables (ncV) the average frequencies across all replicates and variables in the respective group are shown.

The selection frequencies of SMD, AIR, and MIR for the different variables and variable groups are shown in Fig. 4. SMD with 5, 10, and 20 surrogate variables (red circles, squares, and diamonds) shows comparatively high selection frequencies for all relevant variables reaching 100% for *X*_1_ and *X*_2_, more than 90% for *X*_3_, *X*_4_, *X*_5_, *X*_6_, and c*X*_1_, as well as between 80% and 95% and 40% and 60% for c*X*_2_ and c*X*_3_, respectively. However, also the correlated non-relevant variables are frequently selected here, *X*_7_ and *X*_8_ even with frequencies almost reaching 100%. For MIR with 5, 10, and 20 surrogate variables (blue circles, squares, and diamonds), the selection frequencies range between 86% and 98%, while for SMD and MIR with 100 surrogate variables (red and blue triangles), the selection frequencies show similar values below 80% for *X*_3_, *X*_4_, *X*_5_, and *X*_6_ and around 90% and 80% for *X*_1_ and *X*_2_, respectively. For c*X*_2_ and c*X*_3_, MIR shows selection frequencies of around 60% and 15% independent from the number of surrogate variables used, while the frequencies of SMD are much more dependent on this parameter. This is also evident for the non-relevant variables, as MIR has similarly low frequencies, while SMD is characterized by higher, more variable frequencies.

For AIR (black triangle in Fig. 4), selection frequencies are lower than for MIR and SMD (red and blue symbols) when correlations between the variables exist. This applies for the causal variables, where the values for *X*_1_, c*X*_1_, and c*X*_2_ are at around 80%, 65%, and 25%, respectively, and for the non-causal variables that show values at or close to zero for all variables.

A comparison of different *P*-value thresholds for MIR and AIR shows that the used value of .01, the default value of AIR, is reasonable (Supplementary Fig. S15). The comparison of the selection of related variables by SMD and MIR shows no significant differences, which is due to the fact that this study does not use variables that exhibit the biases outlined above (Supplementary Fig. S16). The MFI values used for the selection of related variables are additionally shown in Supplementary Fig. S17.

The average run times for AIR, SMD, and MIR were around 25 ms, between 25 and 40 s, and between 60 and 90 s, respectively (run times were longer when larger values for *s* were used).

From the correlation study, it can be concluded that MIR is more powerful for correlated variables than AIR and less sensitive to changes in the number of surrogate variables used than SMD. However, too high values for *s* lead to an increased FPR, which is apparent for the non-causal variables (ncV) in Fig. 4 and the null scenario (Supplementary Fig. S18).

#### 3.1.4 Realistic study

**Figure 5.**
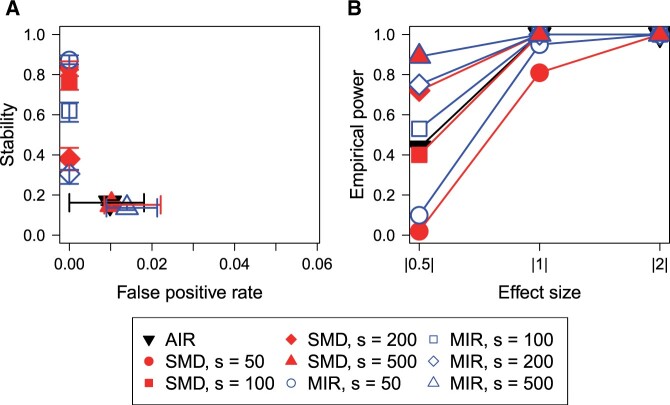
Results of the realistic study: comparison of the performance of AIR, MIR (*P*-value threshold for both: .01) and SMD using different numbers of surrogates. Median, as well as the interquartile range over all 50 replicates for stability and false positive rate (A) and median frequencies of the empirical power for the different effect sizes (B) are shown. The classification error is zero for all investigations.

Figure 5 displays the results for the realistic simulation study. MIR and SMD both show increasing empirical powers as the number of surrogate variables *s* increases (blue and red symbols in Fig. 5B). However, for each value of *s*, MIR has slightly higher empirical powers for variables with low effect sizes (|0.5|). For the medium effect size of |1|, this is only apparent for *s *=* *50 because empirical powers of one are achieved for higher values of *s* (blue and red circles in Fig. 5B). The stability shows an opposite influence of this parameter, since high values are obtained when *s* is low and vice visa. When 100 and 200 surrogate variables are used, SMD shows higher stabilities than MIR almost reaching 0.8 and 0.4, respectively (squares and diamonds in Fig. 5A).

AIR shows similar empirical powers as SMD with *s *=* *100 (red square and black triangle in Fig. 5B). However, the stability is much lower and, just as in SMD and MIR with *s *=* *500 (red and blue triangles in Fig. 5A), the FPR is higher than zero.

The average run times for AIR, SMD, and MIR were 20 s, between 6 and 9 min, and between 22 and 66 min, respectively (run times were longer when larger values for *s* were used).

#### 3.1.5 Real data application

**Figure 6.**
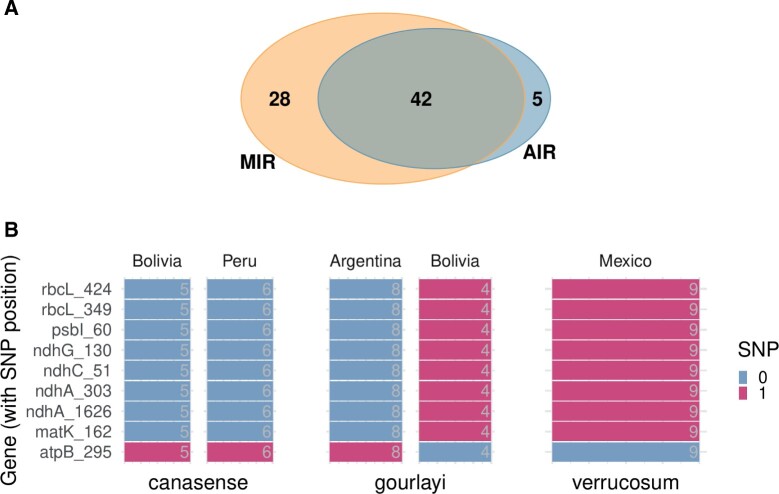
Results from real data application: (A) Venn diagram showing the number and overlap of variables selected by MIR and AIR. (B) Variables from cluster D (see Supplementary Fig. S20) with the SNP distribution (0 = reference and 1 = alternative) for the *Solanum* sect. *Petota* species *canasense*, *gourlayi*, and *verrucosum* and their countries of origin.

The 257 individual SNPs extracted from the multiple sequence alignments are shown in Supplementary Table S2 and RF classification of the species and provenance resulted in accuracies of 94% and 84%, respectively, demonstrating that class specific SNPs are indeed present in the dataset (confusion matrices are shown in Supplementary Tables S3 and S4). To select those SNPs, AIR and MIR were applied and the overlap of selected variables for the classification of the species is shown in Fig. 6A. The majority of variables were selected by both methods but MIR selected 28 variables that were not selected by AIR. Those SNPs were only selected if *s* was set to a comparatively high value of at least 10% of the total variables (see Supplementary Fig. S19).

A heatmap was generated to show the relations of the selected MIR variables (Supplementary Fig. S20). The highly related variables of the single clusters are shown in detail in Fig. 6B (cluster D in Supplementary Fig. S19) as well as in Supplementary Figs S21 (cluster A), S22 (cluster B), and S23 (cluster C). Clusters A, B, and C show SNPs that separate samples of the *canasense* or the *verrucosum* species from the other two species. The SNPs shown in Fig. 6B obviously contain the same information for separating *canasense* and *verrucosum* species, while for *gourlayi* the SNPs are inconclusive. However, the selected SNPs are also applicable to separate the provenance of the *gourlayi* species. Both clusters, C and D, separate subgroups based on the provenance of the *canasense* or *gourlayi* species and contain variables that are exclusively selected by MIR.

## 4 Discussion and conclusion

In this study, we introduced two novel approaches that are based on surrogate variables in RF: MFI for relation analysis and MIR as a feature selection approach considering feature relations. We demonstrated that both, MFI and MIR are not biased regarding variables with different number of categories and category frequencies. They can be applied in RF classification, regression, and survival analyses for the selection of related and important features based on *P*-values obtained by the testing procedure of Janitza *et al.* (2018).

The approaches presented here should be viewed in the context of the challenge of correlated variables for the interpretation of RF models. It is know that the frequently applied permutation importance overestimates the importance of correlated features because all dependencies, including those between variables, are broken by the permutation forcing the model to extrapolate to data regions with little to no data (Hooker *et al.* 2021). To correct for this characteristic, conditional approaches have been developed (Strobl *et al.* 2008, Debeer and Strobl 2020, Watson and Wright 2021, Molnar *et al.* 2023). They evaluate variables in dependence with each other, which is often computationally and time consuming, especially when analysing high-dimensional data. Variable importance scores based on surrogate variables, such as the method presented here, take a different approach to consider correlations for variable importance, as they deliberately reinforce this dependence to improve variable selection performance. The comparison of MIR with AIR in this publication demonstrates this because a higher stability and power is obtained, especially for variables containing similar information. However, it may happen that uncorrelated variables with low but existing impact on the model are selected by AIR and not by MIR. For a comprehensive analysis of the involved variables, assessing them individually and together with other variables, AIR and MIR could be applied together and MFI could subsequently be used to analyse the relationships between all selected variables to obtain a complete picture.

The comparison of MIR with SMD showed lower probabilities of false selections and a smaller sensitivity to changes of the crucial parameter *s*, which determines the number of surrogates used (Seifert *et al.* 2019). Since this parameter is crucial to achieve an optimal compromise between high power and low false selections, a smaller sensitivity to this parameter enables the usage of higher values resulting in a higher power. However, we have shown that even for MIR, too high values for *s* should not be used and additional analyses of data with different numbers of features and correlation structures are needed to find optimal values for the specific data types. We currently recommend for high-dimensional settings to use 1–2% of the total variables as utilized number of surrogate variables in combination with a *P*-value threshold of .01. However, for data with comparatively few variables, higher values for *s*, e.g. 10% of the total variables, could be advantageous. We have shown this here by the analysis of a real dataset for which variables of high interest for interpretation were specifically selected by MIR, but only when sufficiently high numbers of *s* were used.

Due to the non-existing biases, a wide application of MFI and MIR to data with different distributions, such as continuous (e.g. metabolomics, proteomics, and isotopolomics), categorical (genomics), or proportional data (epigenomics) is possible. MFI is especially promising for the relation analysis of features across different datasets, in which, in addition to different omics levels, even phenotypic information and clinical biomarkers could be included. In addition, omics data could be combined with data from spectroscopic experiments, for example based on infrared, Raman, or X-ray radiation.

MFI is a relation parameter based on the performance similarity of two variables at many individual splits in a RF model. Since this model is obtained by a greedy algorithm, also MFI does not consider more complex interactions between several variables. These could, however, be addressed by the combination of surrogate variables with complex split procedures, such as those used in diversity forests (Hornung 2022). Nevertheless, MFI allows the selected features to be divided into groups with similar impact on the RF model. These groups and the feature relations they are based on could be linked to known biological interactions, for example in metabolic pathways, in a similar way as recently conducted with SMD (Wenck *et al.* 2022). In subsequent analyses, the groups of related features could be utilized instead of individual features to robustly identify samples with specific properties, e.g. by applying pathway-based approaches (Seifert *et al.* 2020). In addition, based on MFI, subgroups of samples could be revealed by the identification of subgroup specific features. We demonstrated this here by the identification of provenance specific subgroups in the classification model for different *Petota* species. Identified subgroups could subsequently be applied for the characterization of the analysed samples and to improve the machine learning model (Goel *et al.* 2020).

In conclusion, the novel approaches MIR and MFI are very promising for the powerful selection of relevant features and the comprehensive investigation of the complex connection between features and outcome in omics, multi-omics, and other data.

## Supplementary Material

btad471_Supplementary_Data
