# Supplementary material for: Exploitation of surrogate variables in random forests for unbiased analysis of mutual impact and importance of features
Source: Bioinformatics. 2023 Jul 31;39(8):btad471. doi: 10.1093/bioinformatics/btad471 (PMC10403431; doi:10.1093/bioinformatics/btad471)
Supplement: btad471_Supplementary_Data [file btad471_supplementary_data.pdf]

Supplementary information "Exploitation of surrogate variables in random forests for unbiased analysis of mutual impact and importance of features"

Lucas F. Voges, Lukas C. Jarren, Stephan Seifert

Table S 1: Overview of the simulation studies according to the ADEMP scheme

	Bias study	Correlation study	Realistic study
Aim	bias analysis	performance comparison	performance comparison
Data-generation mechanism	null variables with known expression possibilities / MAF with sample/rnorm function	variables with simple correlation structures with simulateModule function	variables with realistic correlation structures using a real covariance matrix
Methods	AIR, SMD, MFI, MIR	AIR, SMD, MFI, MIR	AIR, SMD, MIR
Target	importance, relation	variable selection	variable selection
Performance measures	importance, relation	selection frequency	stability, classification error empirical power, false positive rate

Table S 2: Genes reported together with the position of SNVs/SNPs found by multiple sequence alignment.

gene	position
accD	90, 170, 315, 1479
atpA	209, 210, 568, 606, 1191, 1458
atpB	10, 155, 295, 315, 386
atpF	422, 611, 937, 984
ccsA	595
cemA	139, 651
matK	162, 225, 353, 381, 572, 997, 1001, 1033, 1450
ndhA	91, 184, 303, 331, 650, 676, 798, 818, 868, 1023, 1039, 1229, 1243, 1276, 1576, 1626, 1661, 1921, 1964
ndhC	51, 175
ndhD	495, 534, 570, 1011
ndhF	201, 507, 664, 1407, 1516, 1986, 2031, 2073
ndhG	130, 204
ndhH	369, 591, 837, 879, 1021, 1068
ndhJ	40, 378
petA	8, 78, 666
petB	178, 203, 305, 536, 683, 753, 973, 1042
petD	51, 222, 223, 435, 688, 872
psaA	183, 687, 810, 858, 1287, 2029
psaC	99, 117, 141
psaJ	135
psbA	870
psbB	399, 918, 963, 1125, 1218
psbI	60

gene	position
psbL	84
rbcL	349, 424, 425, 495, 688, 763, 784, 785, 927, 1316, 1345, 1346
rpl14	169
rpl16	115, 128, 325, 682, 822, 861, 921, 1063, 1167, 1215
rpl20	193
rpl22	114, 192, 321
rpl33	183
rpoA	648, 699
rpoB	91, 513, 1022, 1218, 1557, 1759, 2067, 2082
rpoC1	452, 515, 942, 1007, 1044, 1311, 1402, 1423, 1456, 1672, 2430, 2730
rpoC2	808, 987, 1147, 1338, 1541, 2322, 2737, 2751, 2940, 3126, 3154, 3222, 3750
rps11	18, 165, 230
rps14	171, 294
rps15	37, 54, 240
rps16	642
rps2	108, 210
rps3	141, 207, 309, 363, 593, 627, 650
rps4	116, 441, 505
ycf1	225, 514, 1079, 1080, 1230, 1293, 1353, 1437, 1457, 1460, 1551, 1587, 1591, 1642, 1684, 1741, 1794, 1810, 2133, 2205, 2337, 2344, 2566, 2703, 2752, 2754, 2916, 3022, 3090, 3114, 3147, 3306, 3332, 3347, 3349, 3513, 3536, 3772, 3840, 3961, 3990, 4150, 4194, 4379, 4481, 4483, 4503, 4610, 4725, 5280, 5281, 5458, 5493, 5517, 5518, 5652
ycf3	10, 49, 727, 796, 1207, 1223, 1239, 1657

Table S 3: Confusion matrix for random forest classification of the *Solanum* sect. *Petota* species from the plastid data subset. Accuracy: 94%, Random forest settings: num.trees = 1000, min.node.size = 1, mtry = $p_3^{3/4}$

	canasense	gourlayi	verrucosum
canasense	10	1	0
gourlayi	0	11	1
verrucosum	0	0	9

Table S 4: Confusion matrix for random forest classification of the *Solanum* sect. *Petota* country of origin from the plastid data subset. Accuracy: 84%, Random forest settings: num.trees = 1000, min.node.size = 1, mtry = $p_3^{3/4}$

	Argentina	Bolivia	Mexico	Peru
Argentina	8	0	0	0
Bolivia	1	6	1	1
Mexico	0	0	9	0
Peru	0	2	0	4

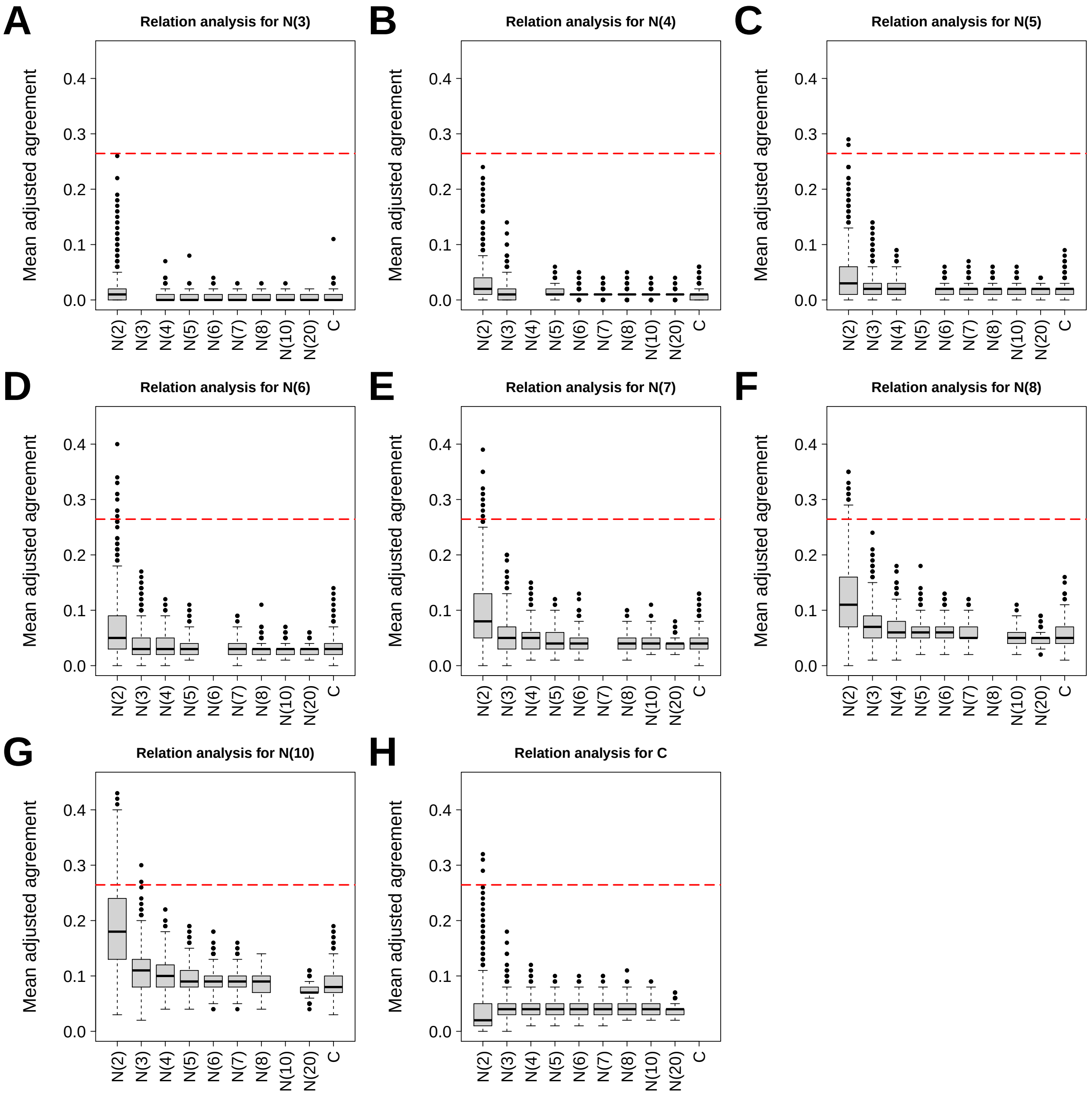

Figure S 1: Null Scenario A (classification): Relation analysis based on mean adjusted agreement for the nominal variables with 3, 4, 5, 6, 7, 8, 10 categories (A-G) and the continuous variable (H). The red line shows the threshold for the selection of related variables. The respective missing values result from the non-existing relations of variables with themself and the relations of the other variables are shown in Figure 1.

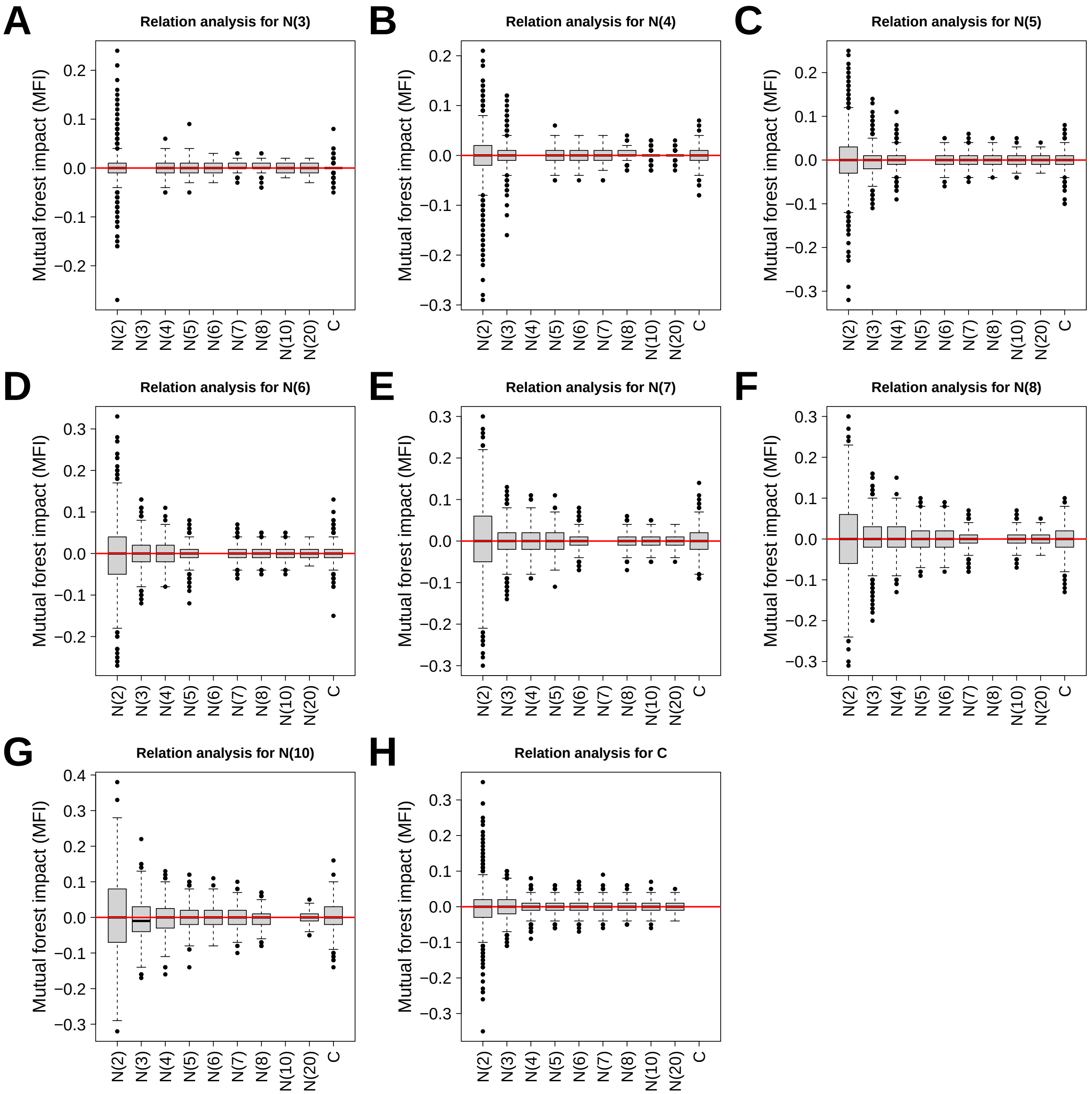

Figure S 2: Null Scenario A (classification): Relation analysis based on MFI for the nominal variables with 3, 4, 5, 6, 7, 8, 10 categories (A-G) and the continuous variable (H). The red line shows a relation value of 0. The respective missing values result from the non-existing relations of variables with themself and the relations of the other variables are shown in Figure 1.

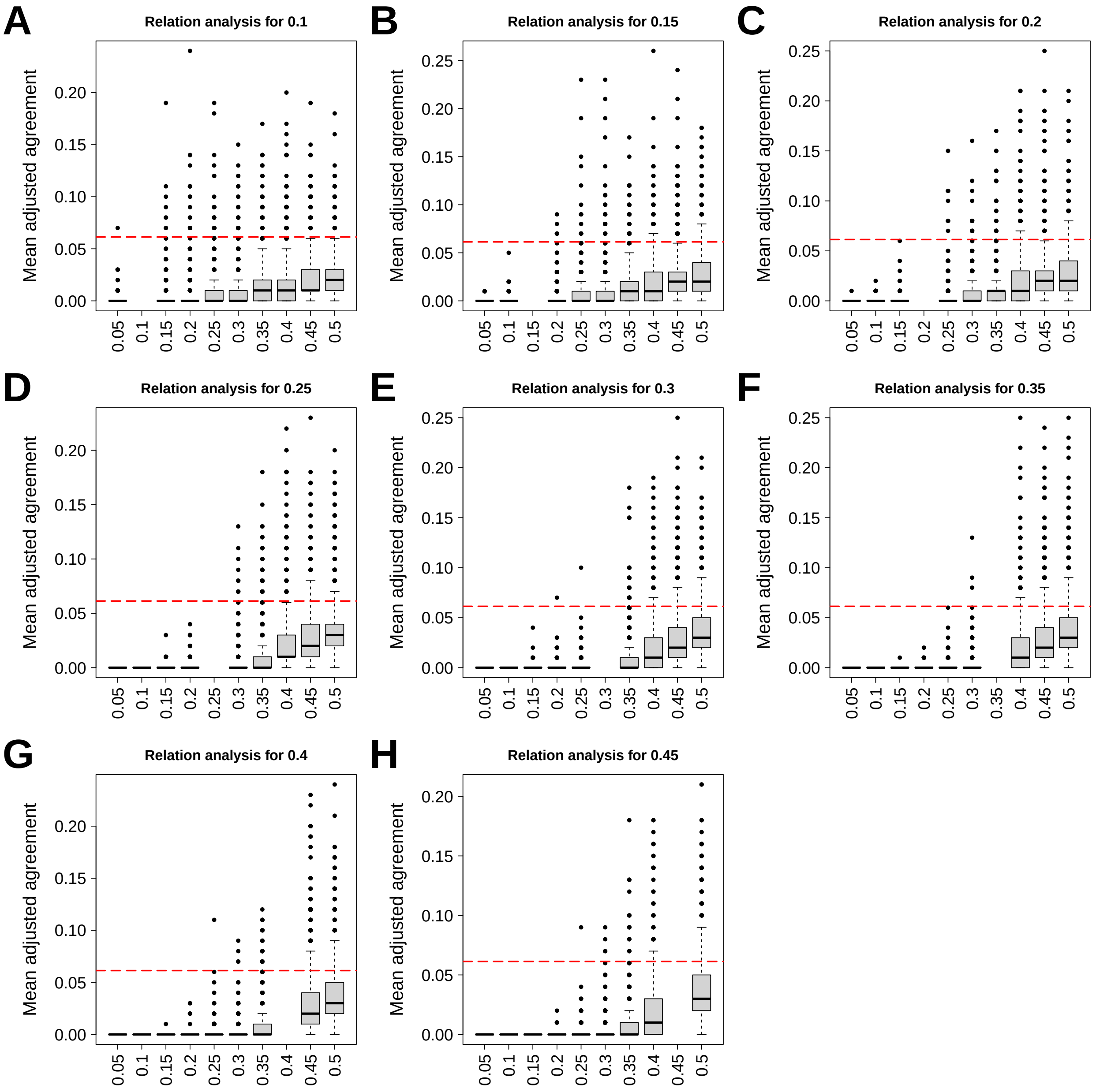

Figure S 3: Null Scenario B (classification): Relation analysis based on mean adjusted agreement for the variables with minor allele frequencies of 0.1 to 0.45. The red line shows the threshold for the selection of related variables. The respective missing values result from the non-existing relations of variables with themself and the relations of the other variables are shown in Figure 2.

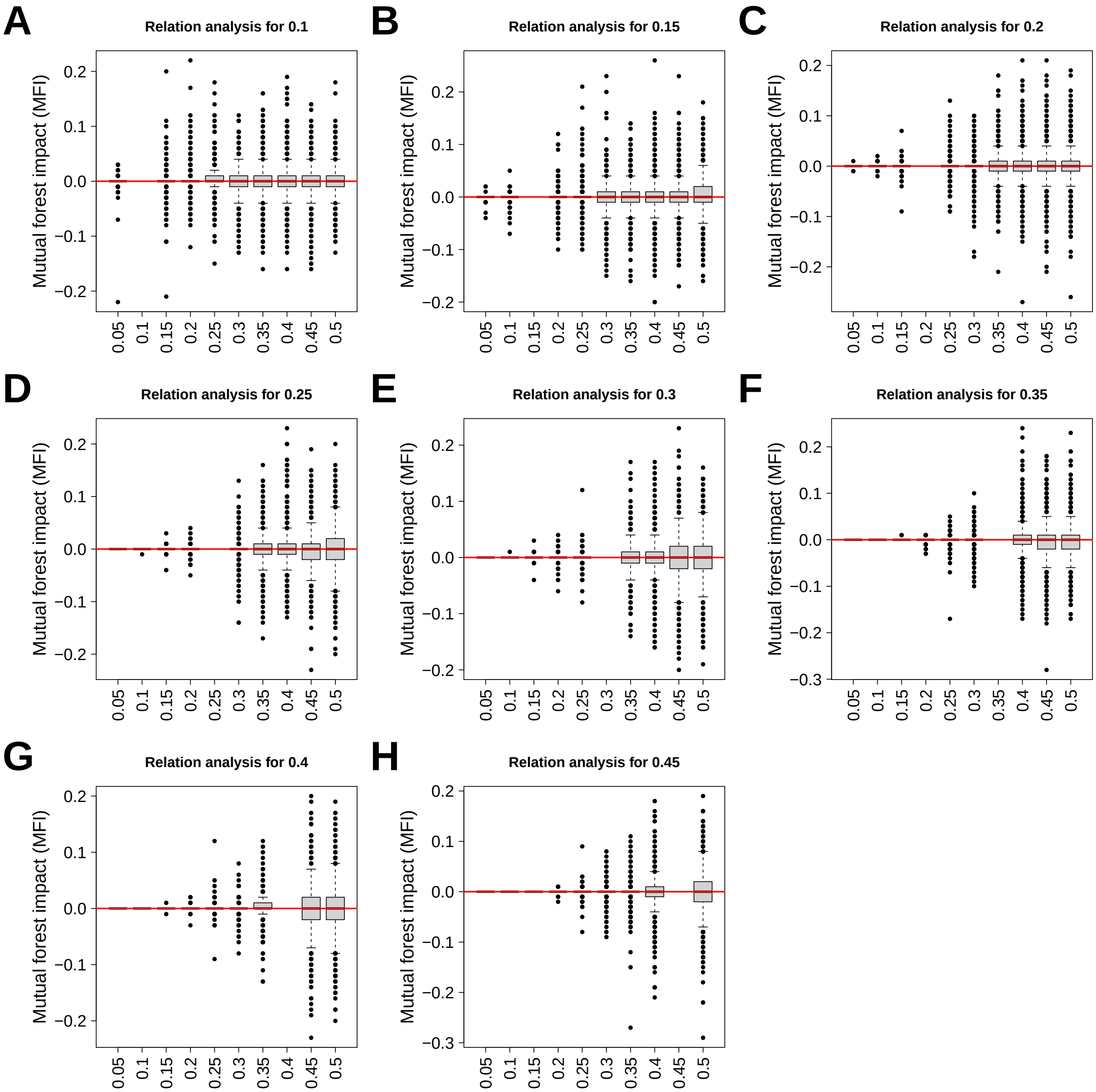

Figure S 4: Null Scenario B (classification): Relation analysis based on MFI for the variables with minor allele frequencies of 0.1 to 0.45. The red line shows a relation value of 0. The respective missing values result from the non-existing relations of variables with themself and the relations of the other variables are shown in Figure 2.

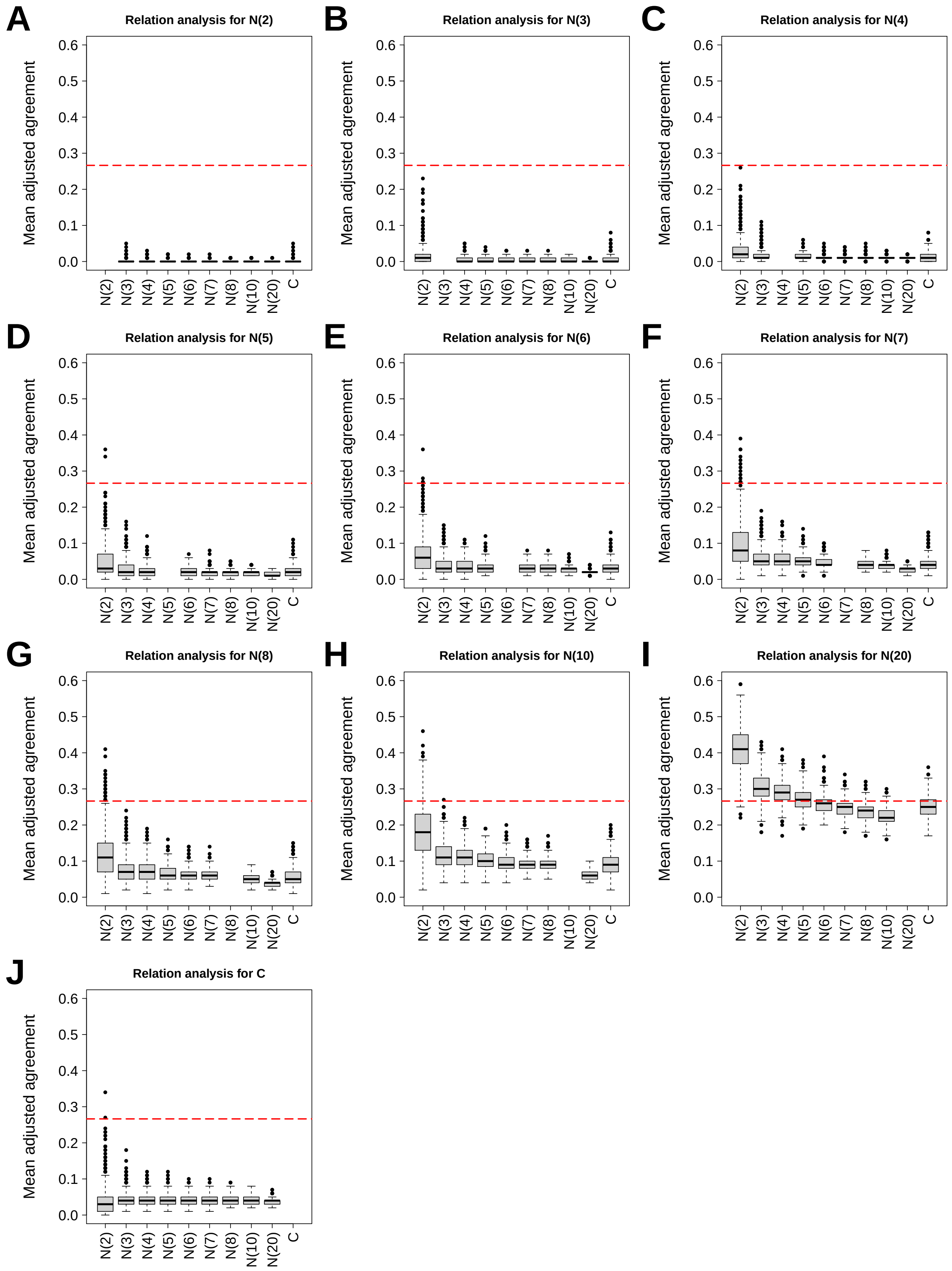

Figure S 5: Null Scenario A (regression): Relation analysis based on mean adjusted agreement for the nominal variables (A-I) and the continuous variable (J). The respective missing values result from the non-existing relations of variables with themself and the red line shows the threshold for the selection of related variables.

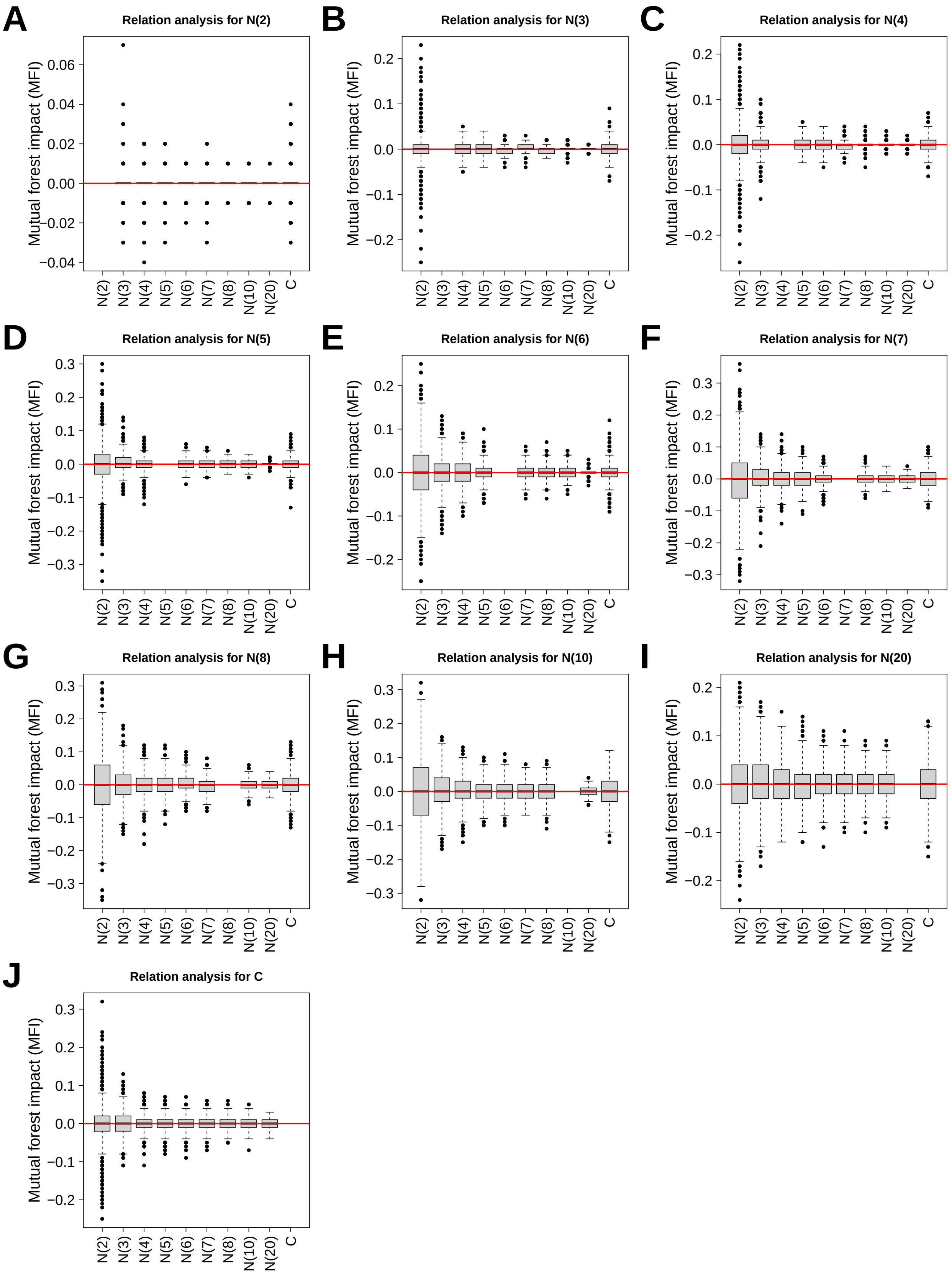

Figure S 6: Null Scenario A (regression): Relation analysis based on MFI for the nominal variables (A-I) and the continuous variable (J). The respective missing values result from the non-existing relations of variables with themself and the red line shows a relation value of 0.

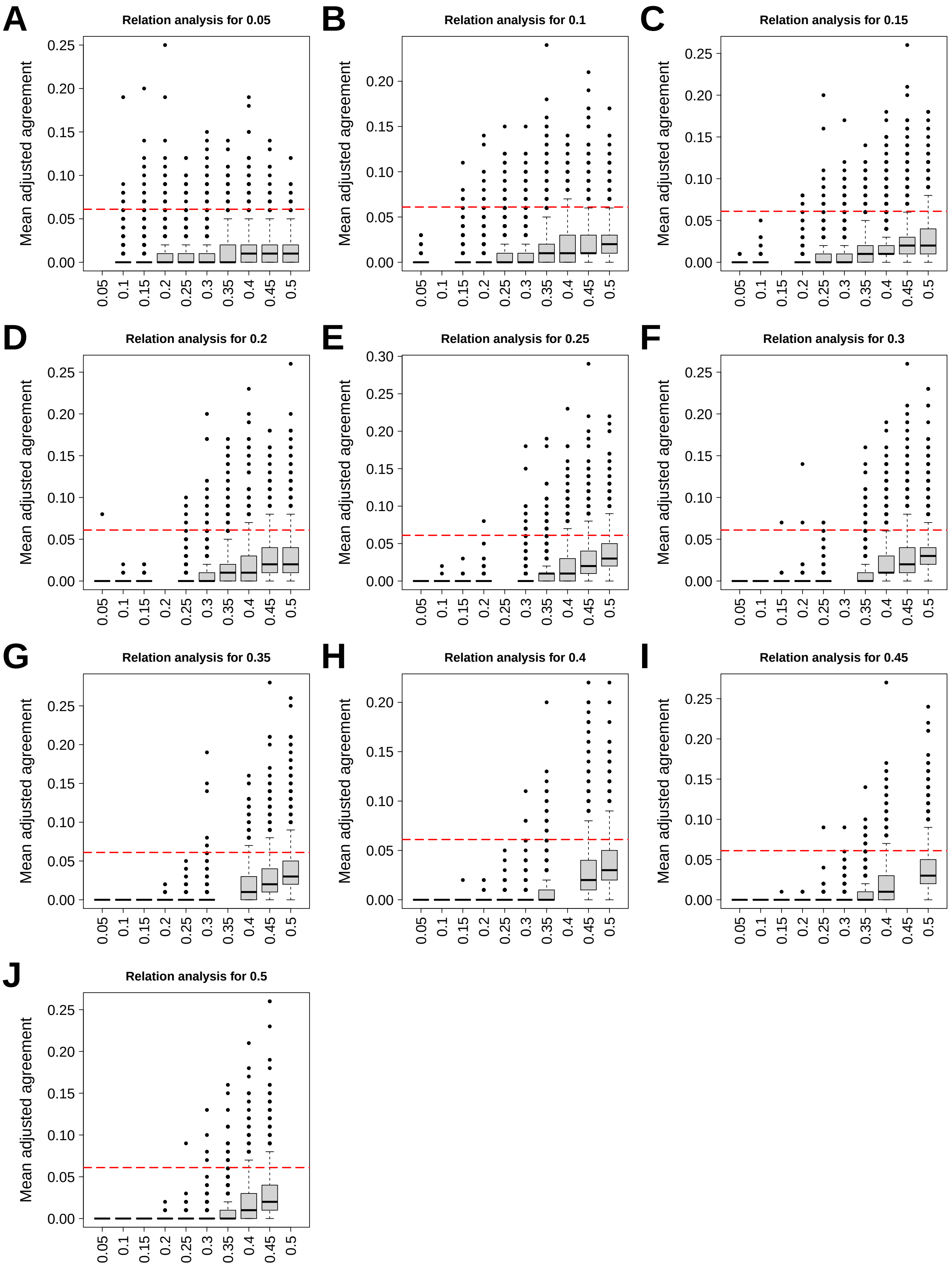

Figure S 7: Null Scenario B (regression): Relation analysis based on mean adjusted agreement for the variables with different minor allele frequencies. The respective missing values result from the non-existing relations of variables with themself and the red line shows the threshold for the selection of related variables.

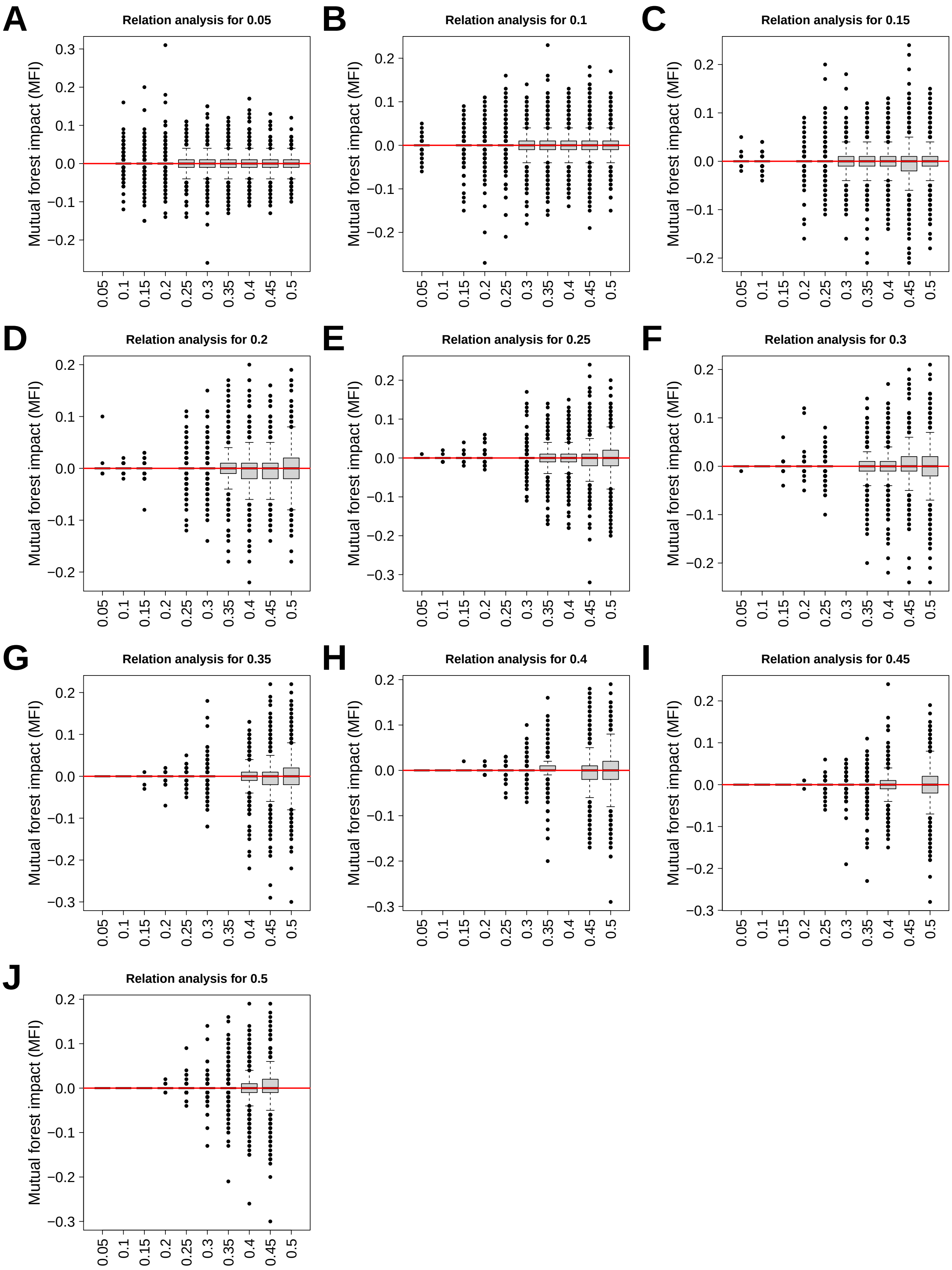

Figure S 8: Null Scenario B (regression): Relation analysis based on MFI for the variables with different minor allele frequencies. The respective missing values result from the non-existing relations of variables with themself and the red line shows a relation value of 0.

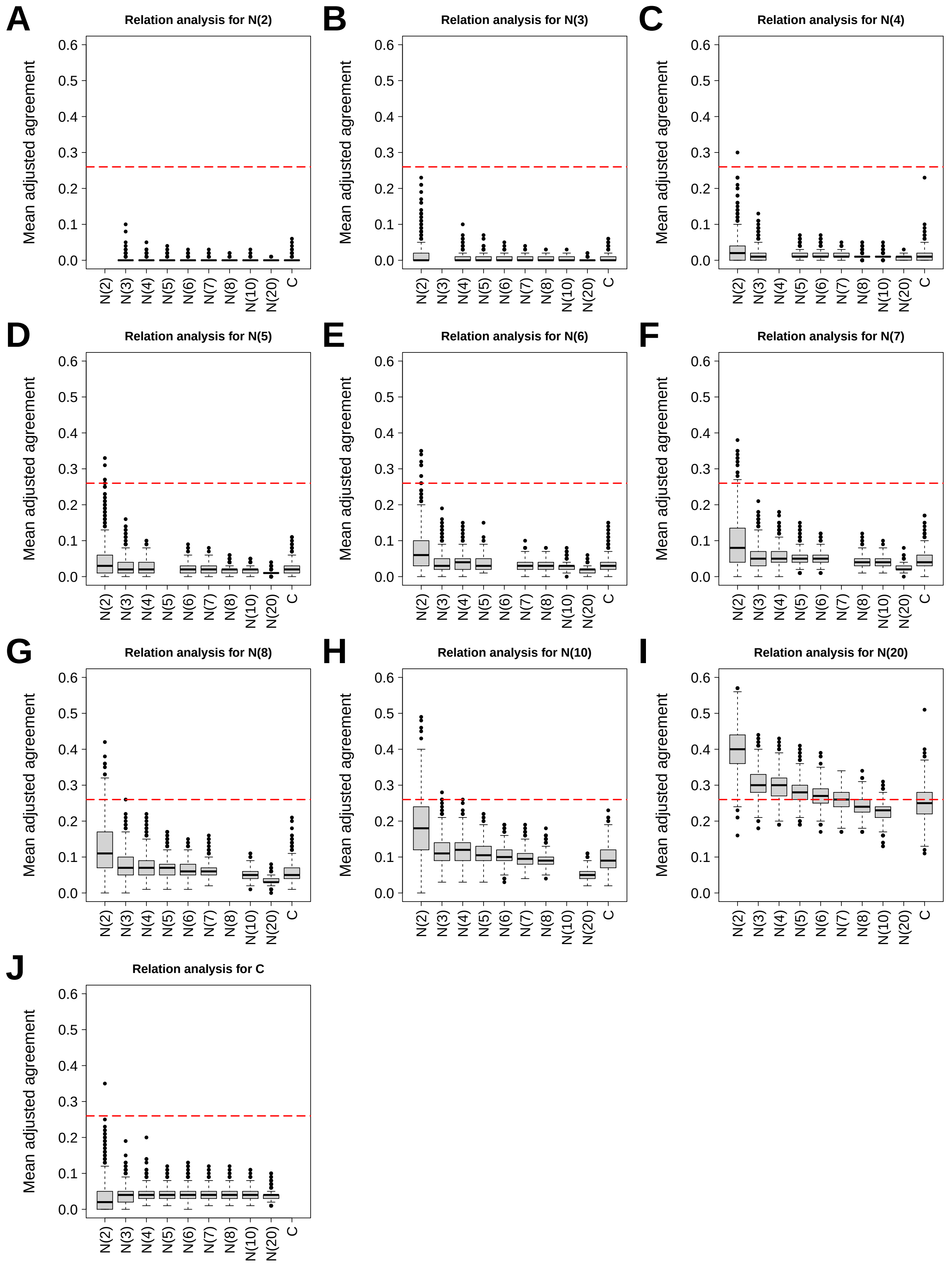

Figure S 9: Null Scenario A (survival): Relation analysis based on mean adjusted agreement for the nominal variables (A-I) and the continuous variable (J). The respective missing values result from the non-existing relations of variables with themself and the red line shows the threshold for the selection of related variables.

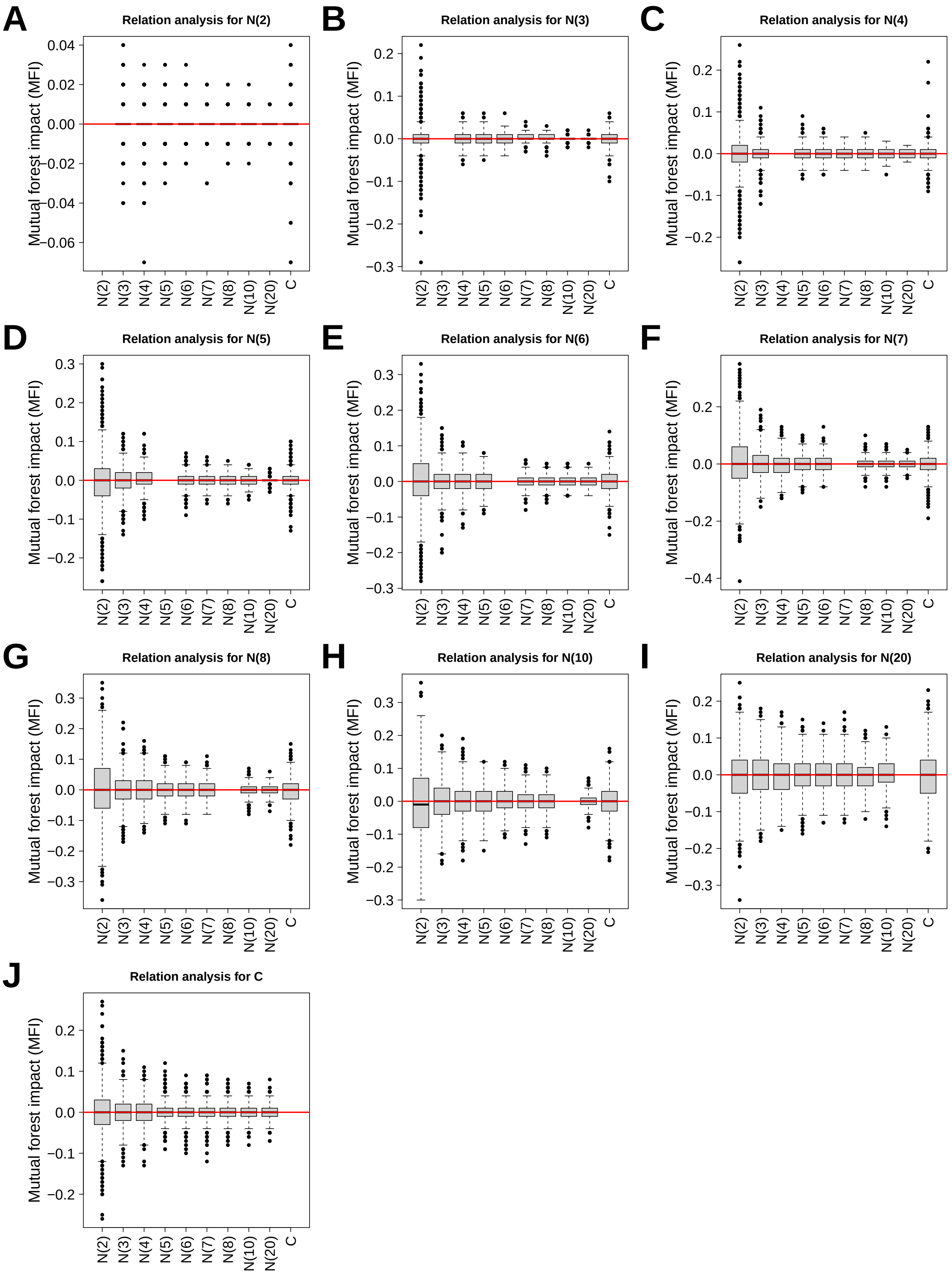

Figure S 10: Null Scenario A (survival): Relation analysis based on MFI for the nominal variables (A-I) and the continuous variable (J). The respective missing values result from the non-existing relations of variables with themself and the red line shows a relation value of 0.

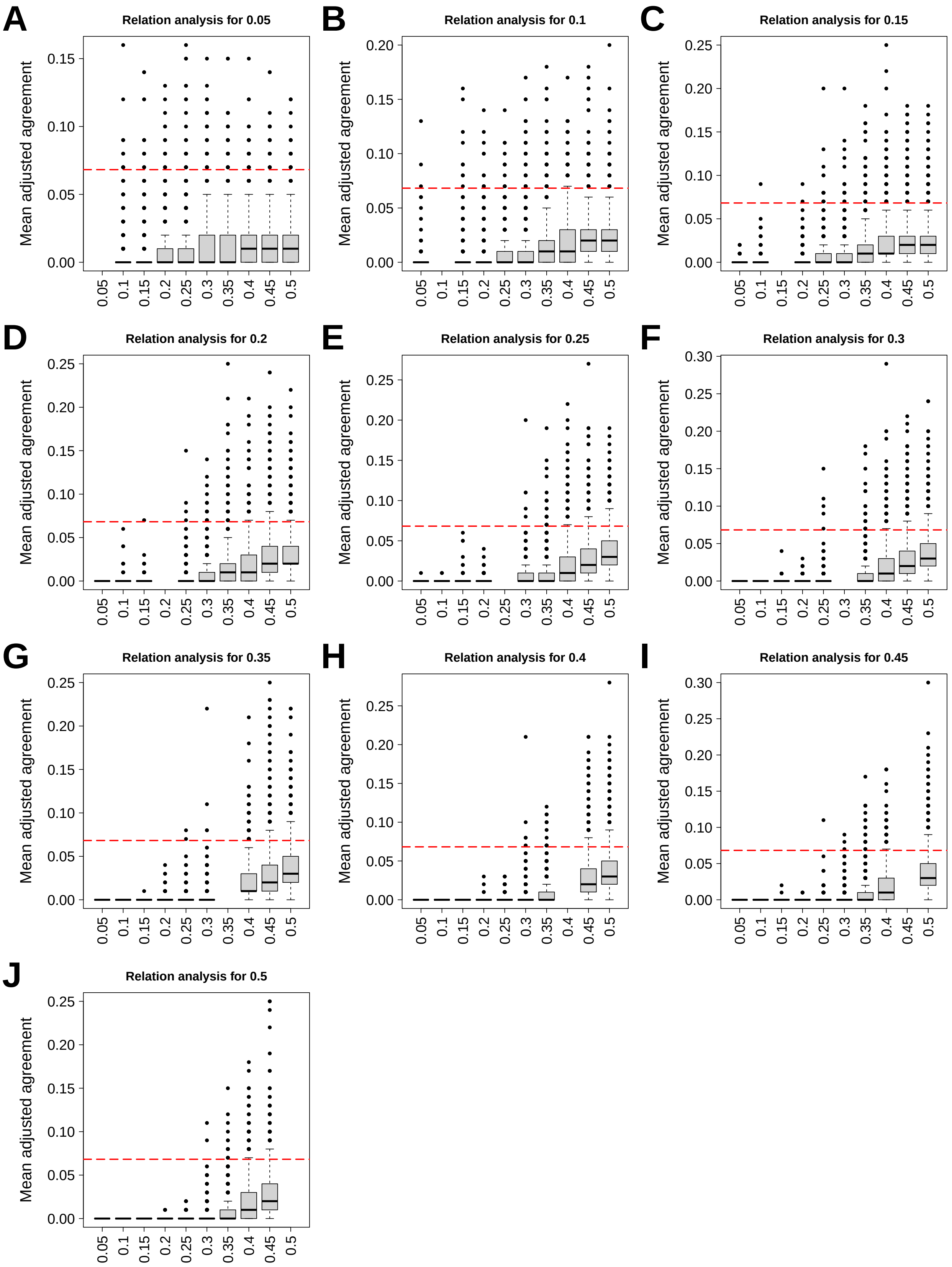

Figure S 11: Null Scenario B (survival): Relation analysis based on mean adjusted agreement for the variables with different minor allele frequencies. The respective missing values result from the non-existing relations of variables with themself and the red line shows the threshold for the selection of related variables.

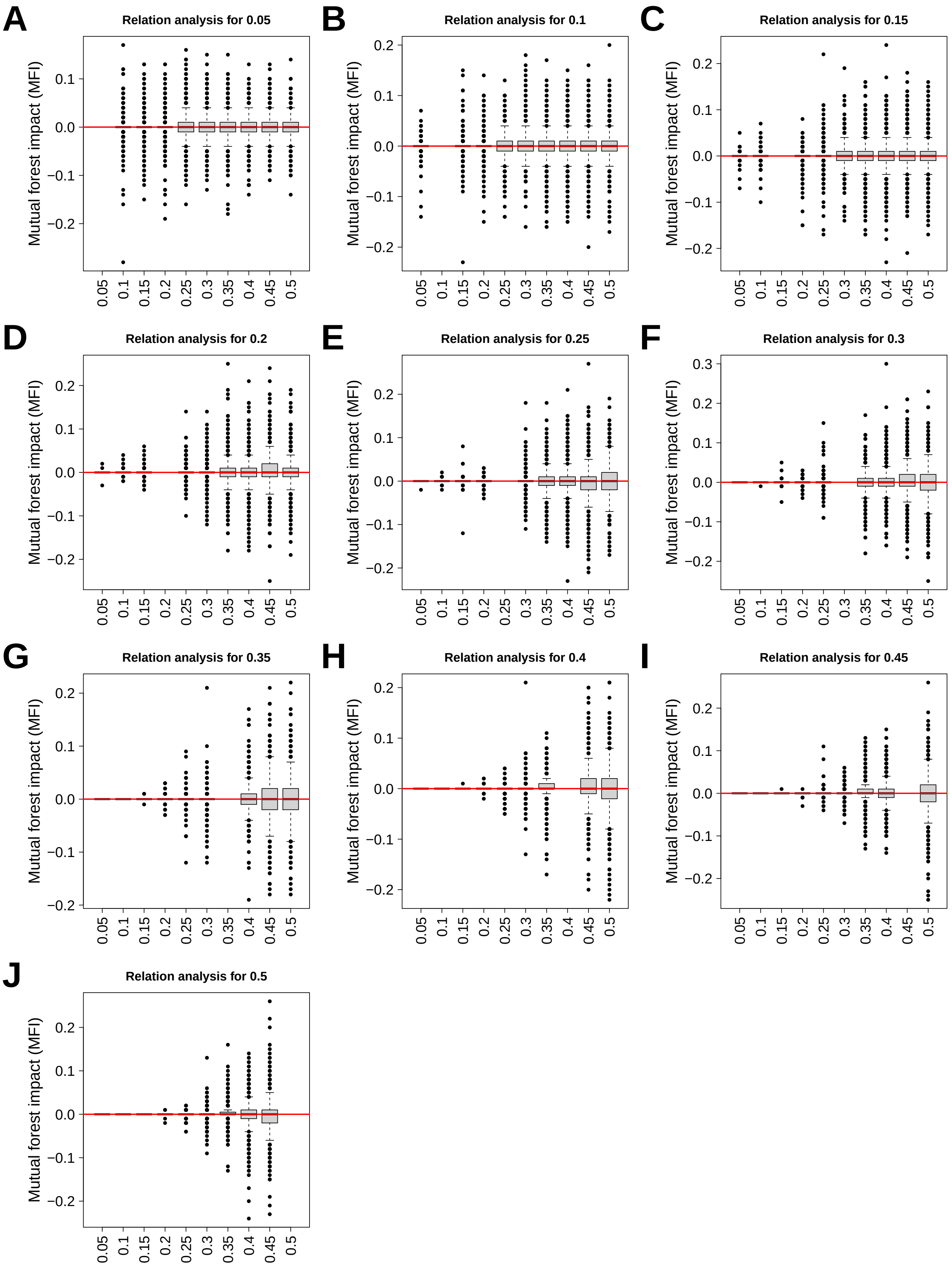

Figure S 12: Null Scenario B (survival): Relation analysis based on MFI for the variables with different minor allele frequencies. The respective missing values result from the non-existing relations of variables with themself and the red line shows a relation value of 0.

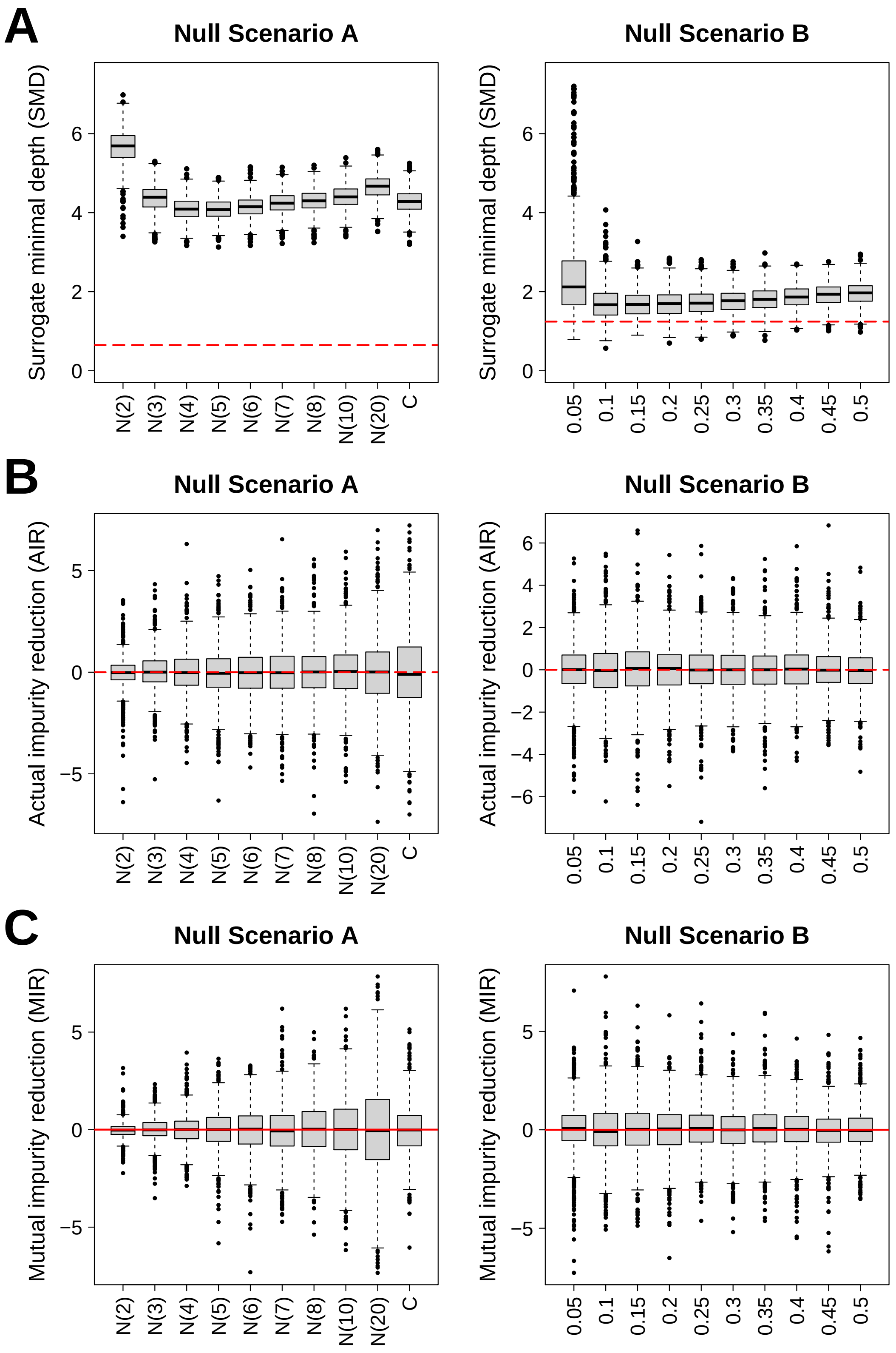

Figure S 13: Variable importances for Null scenarios (regression): Results of SMD (A), AIR (B), and MIR (C) in Null Scenario A (left) and B (right). The respective missing values result from the non-existing relations of variables with themself and the red line shows the threshold for the feature selection and an importance value of 0 for SMD (t=5) and AIR and MIR, respectively.

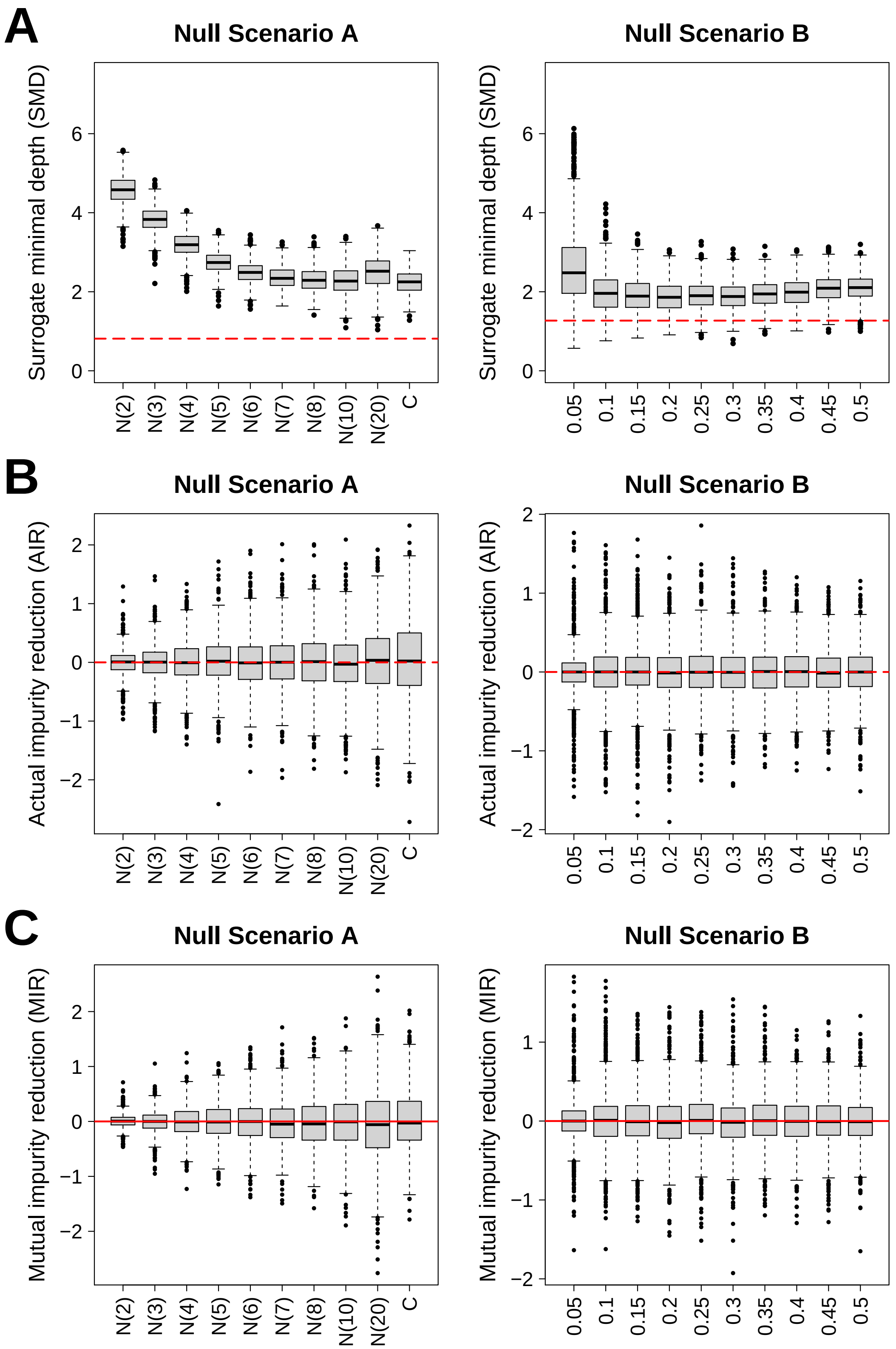

Figure S 14: Variable importances for Null scenarios (survival): Results of SMD (A), AIR (B), and MIR (C) in Null Scenario A (left) and B (right). The respective missing values result from the non-existing relations of variables with themself and the red line shows the threshold for the feature selection and an importance value of 0 for SMD (t=5) and AIR and MIR, respectively.

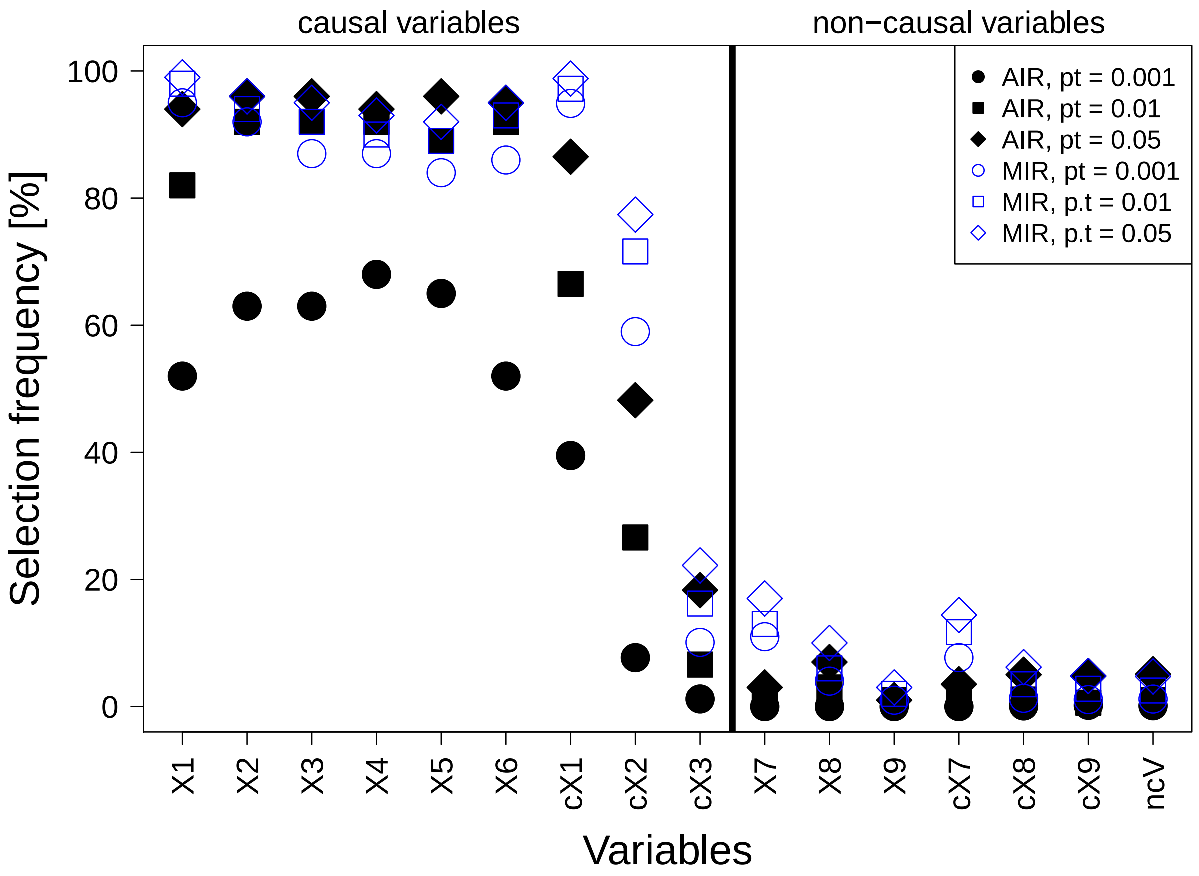

Figure S 15: Results of the correlation study utilizing different numbers p-value thresholds for MIR and AIR. For the basic variables, ($X_1 - X_9$) the selection frequencies are averaged across all 100 replicates, whereas for the six groups of correlated variables, (cX_1 − cX_3 and cX_7 − cX_9) as well as the non-causal variables (ncVs) the average frequencies across all replicates and variables in the respective group are shown.

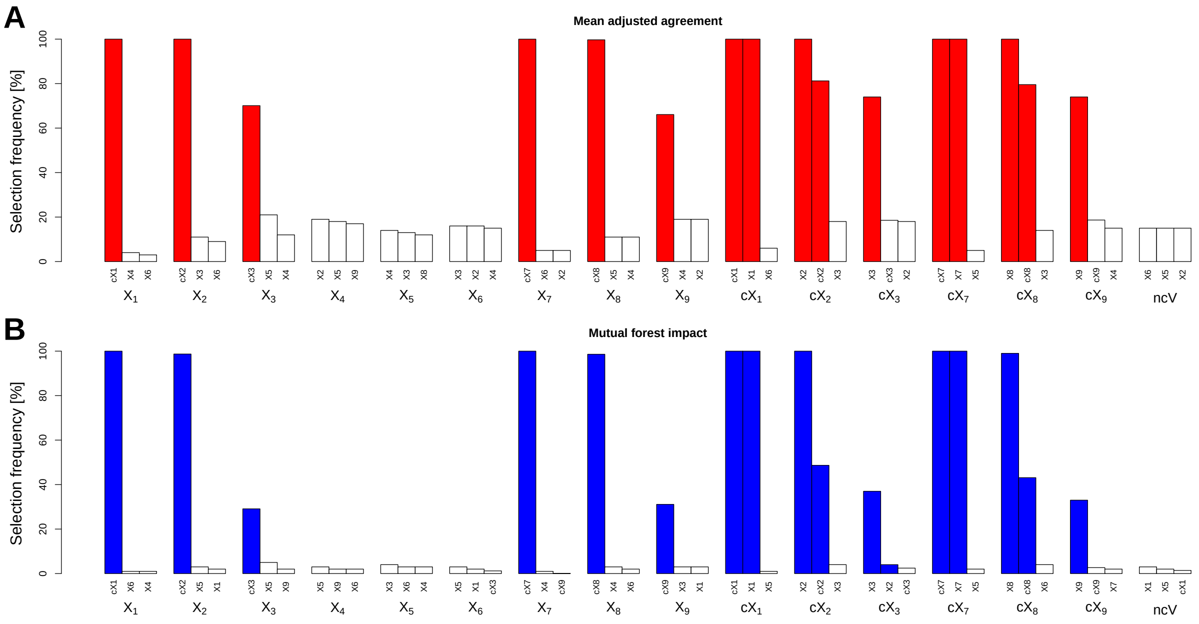

Figure S 16: Variable relation selection frequencies based on mean adjusted ageement (A) and mutual forest impact (B) utilizing an s-value of 10, as well as a t-value of 5 and a p-value threshold of 0.01, respectively. Results for the basic variables ($X_1 - X_9$) and the first variable of each group of variables (cX_1, cX_2, cX_3, cX_6, cX_7, cX_9) are shown. For each variable, the three most often selected variables are shown and the different groups were summarized in one plot. The bars of the correlated variables are colored in red and blue for SMD and MIR, respectively.

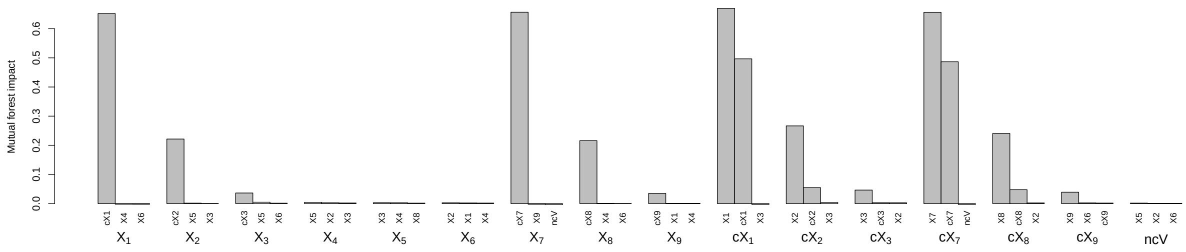

Figure S 17: Averaged mutual forest impact values for the correlation study utilizing an s-value of 10. Results for the basic variables ($X_1 - X_9$) and the first variable of each group of variables (cX_1, cX_2, cX_3, cX_6, cX_7, cX_9) are shown. For each variable, the three variables or variable groups with the highest mean value for mutual forest impact are shown and the bars of the correlated variables are colored in gray.

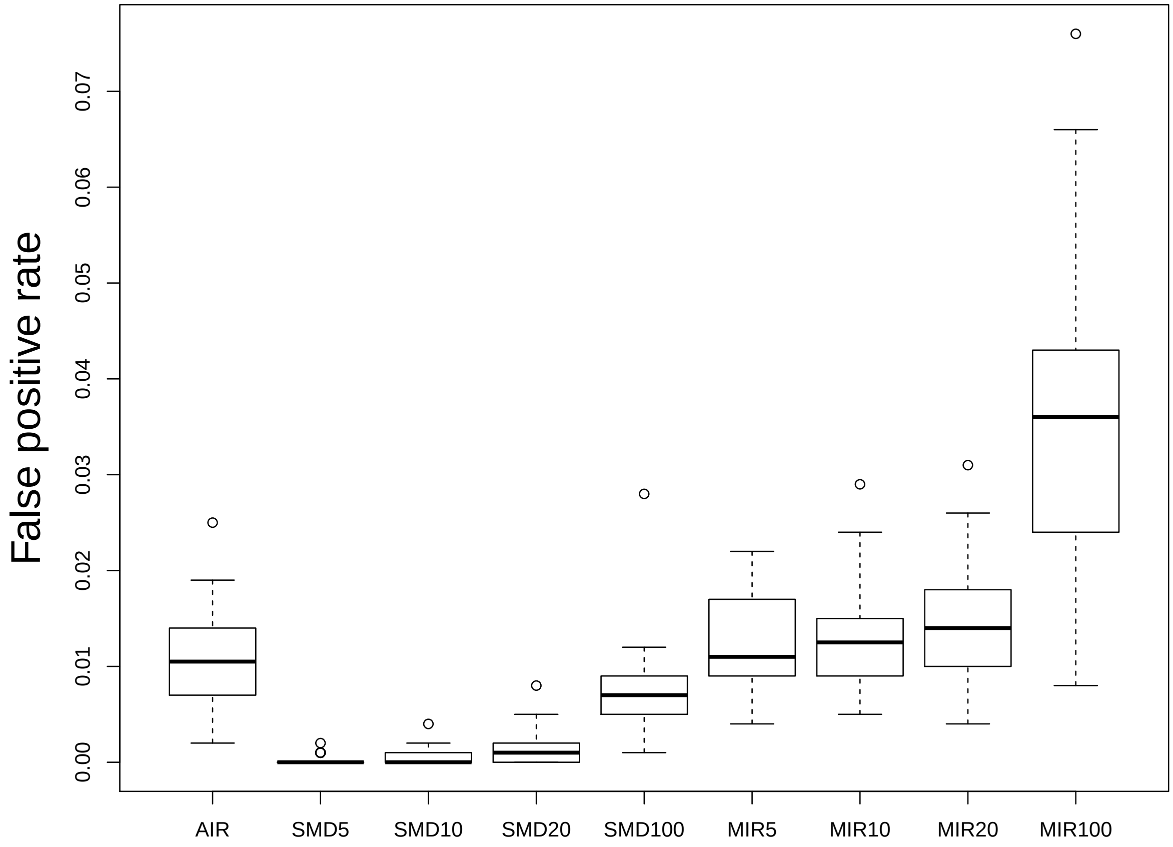

Figure S 18: Results of the null scenario. AIR and MIR were applied with a p-value threshold of 0.01 and SMD and MIR with different numbers of surrogates specified below the respective boxplot

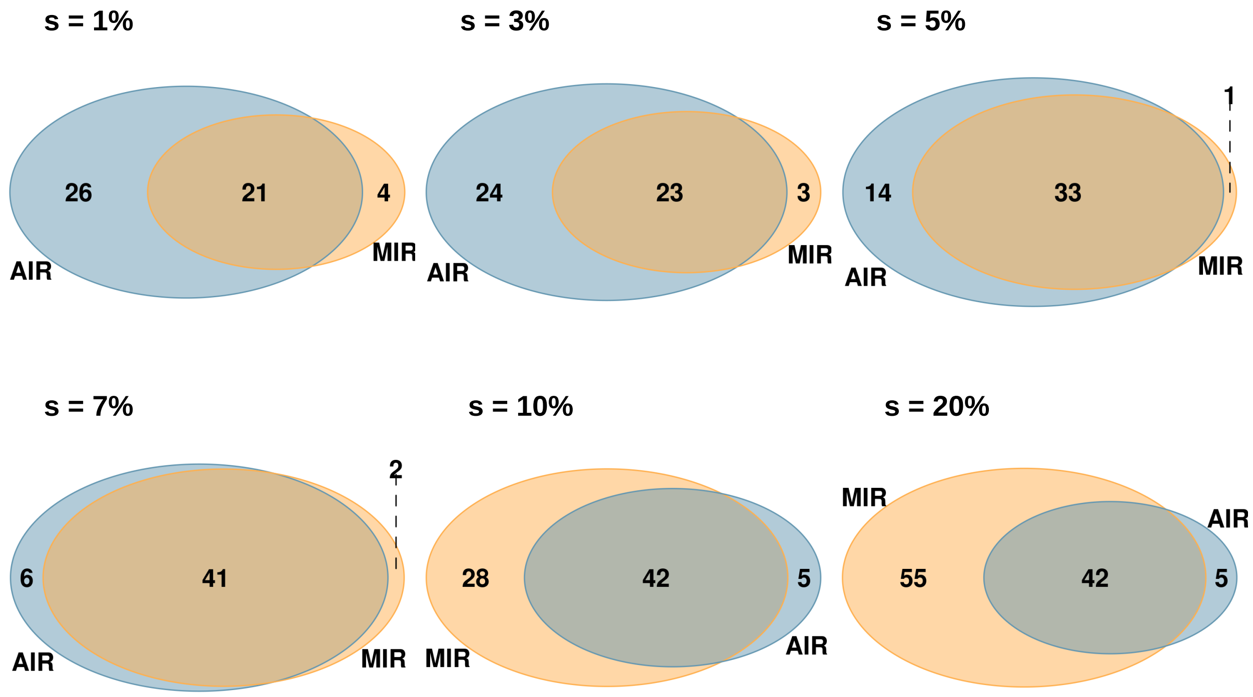

Figure S 19: Venn diagrams showing the number and overlap of variables selected by MIR and AIR in the real data application. 1, 3, 7, 5, 10 and 20% of features ($\widehat{=}$ 3, 8, 13, 18, 26 and 52) used for the surrogate splits (s).

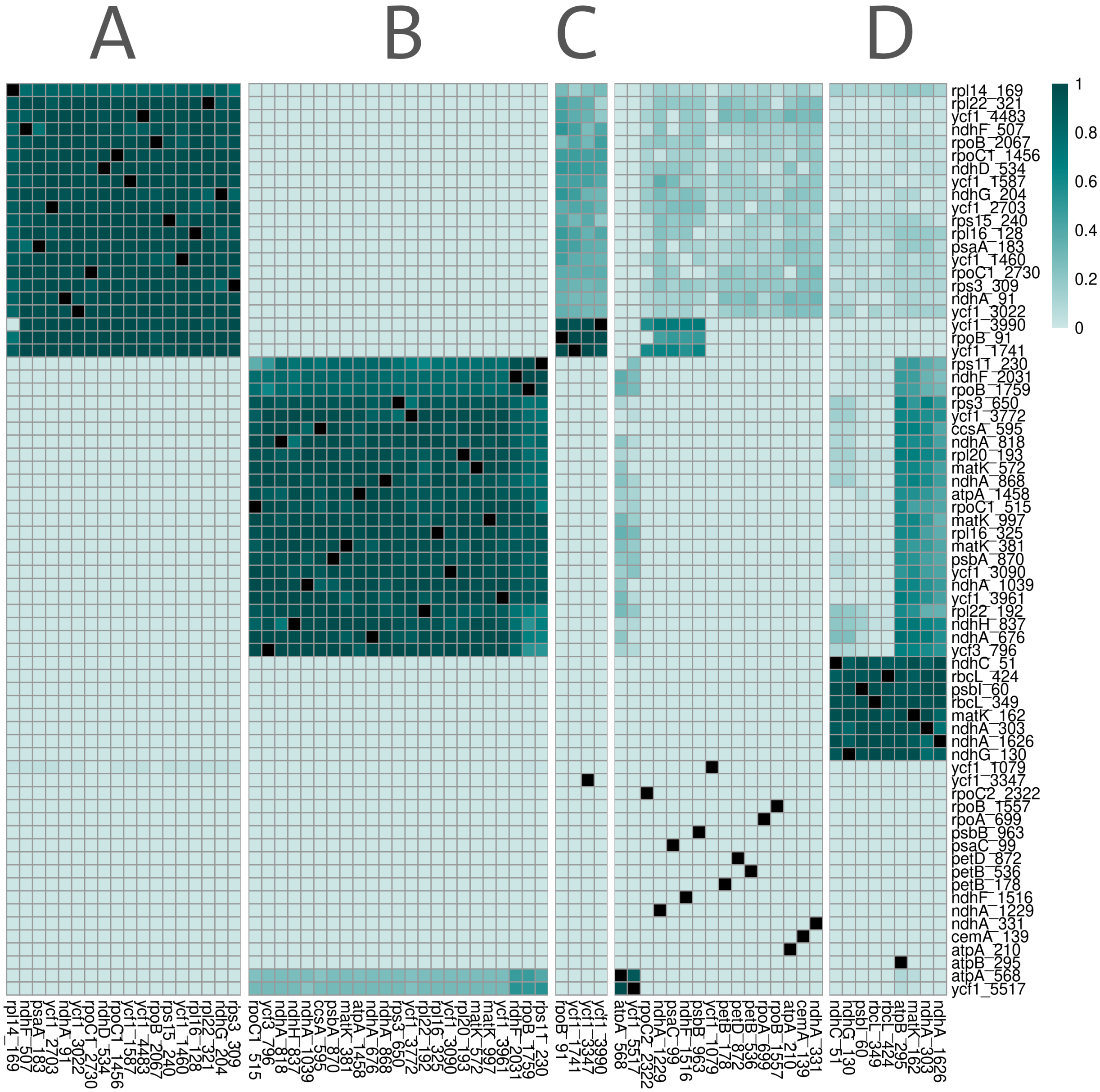

Figure S 20: Heatmap of MIR selected variables from *Solanum* sect. *Petota* plastid data subset. Clustering is initially performed with hierarchical cluster analysis and clusters of variables with high relations are selected (A,B,C,D) columnwise. Variable names describe gene and position of the SNP. Cell colors indicate the relation of the variable pairs; black indicate non existing relations for the variable relation with itself.

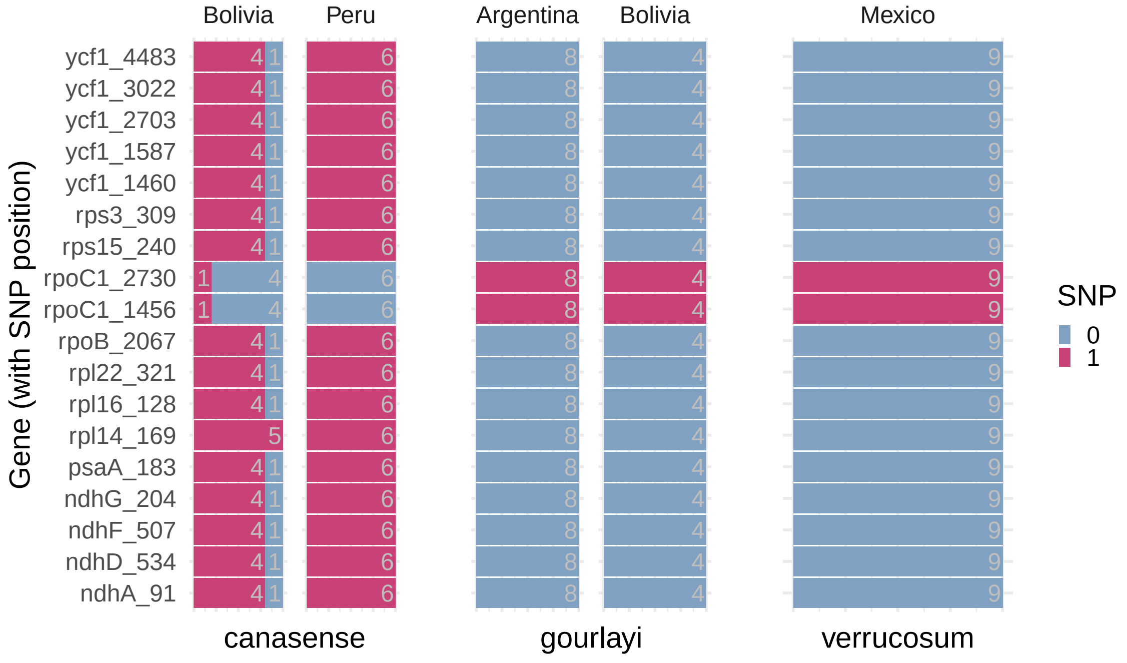

Figure S 21: Variables from cluster **A** (see Figure S 18) with the SNP distribution (0 = reference, 1 = alternative) for the *Solanum* sect. *Petota* species *canasense* (n=11), *gourlayi* (n=12) and *verrucosum* (n=9) and their countries of origin.

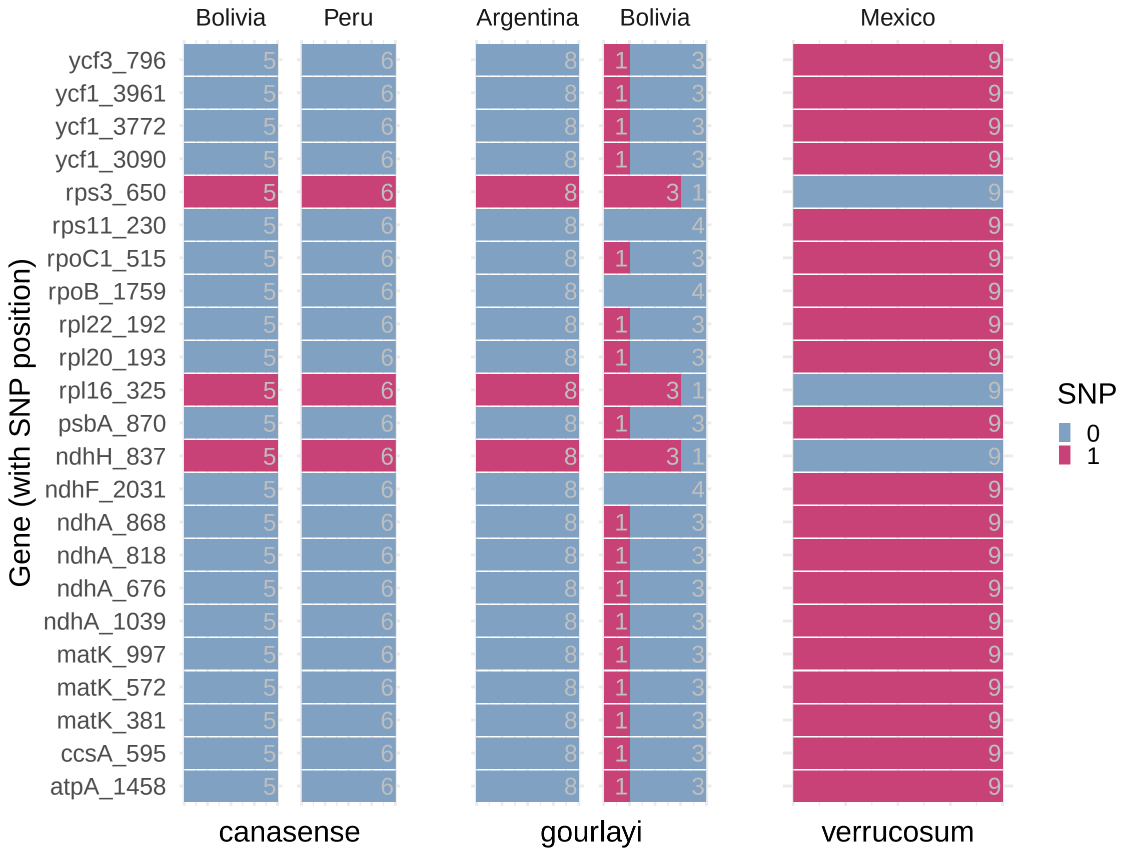

Figure S 22: Variables from cluster **B** (see Figure S 18) with the SNP distribution (0 = reference, 1 = alternative) for the *Solanum* sect. *Petota* species *canasense* (n=11), *gourlayi* (n=12) and *verrucosum* (n=9) and their countries of origin.

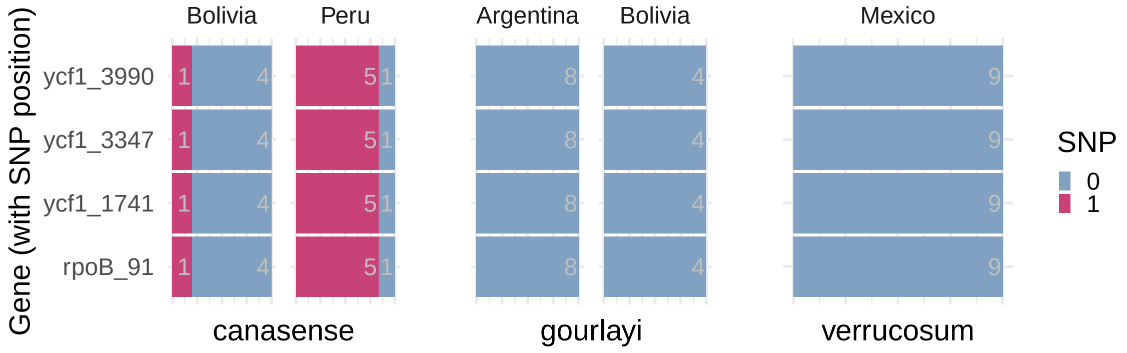

Figure S 23: Variables from cluster **C** (see Figure S 18) with the SNP distribution (0 = reference, 1 = alternative) for the *Solanum* sect. *Petota* species *canasense* (n=11), *gourlayi* (n=12) and *verrucosum* (n=9) and their countries of origin.
